# Identifying optimal substrate classes of membrane transporters

**DOI:** 10.1371/journal.pone.0315330

**Published:** 2024-12-19

**Authors:** Andreas Denger, Volkhard Helms

**Affiliations:** Center for Bioinformatics, Saarland University, Saarbrücken, Germany; UMR-S1134, INSERM, Université Paris Diderot, INTS, FRANCE

## Abstract

Membrane transporters are responsible for moving a wide variety of molecules across biological membranes, making them integral to key biological pathways in all organisms. Identifying all membrane transporters within a (meta-)proteome, along with their specific substrates, provides important information for various research fields, including biotechnology, pharmacology, and metabolomics. Protein datasets are frequently annotated with thousands of molecular functions that form complex networks, often with partial or full redundancy and hierarchical relationships. This complexity, along with the low sample count for more specific functions, makes them unsuitable as classes for supervised learning methods, meaning that the creation of an optimal subset of annotations is required. However, selection of this subset requires extensive manual effort, along with knowledge about the biology behind the respective functions. Here, we present an automated pipeline to address this problem. Unlike previous approaches for reducing redundancy in GO datasets, we employ machine learning to identify a subset of functional annotations in a training dataset. Classes in the resulting predictive model meet four essential criteria: sufficient sample size for training predictive models, minimal redundancy, strong class separability, and relevance to substrate transport. Furthermore, we implemented a pipeline for creating training datasets of transmembrane transporters that cover a wide range of organisms, including plants, bacteria, mammals, and single-cell eukaryotes. For a dataset containing 98.1% of transporters from *S. cerevisiae*, the pipeline automatically reduced the number of functional annotations from 287 to 11 GO terms that could be classified with a median pairwise F1 score of 0.87±0.16. For a meta-organism dataset containing 96% of all transport proteins from *S. cerevisiae*, *A. thaliana*, *E. coli* and human, the number of classes was reduced from 695 to 49, with a median F1 score of 0.92±0.10 between pairs of GO terms. When lowering the percentage of covered proteins down to 67%, the pipeline found a subset of 30 GO terms with a median F1 score of 0.95±0.06.

## Introduction

Proteins fulfill a variety of functions in each cell. One of these functions is the transport of molecules across biological membranes, specifically those molecules that cannot diffuse across the membrane on their own. There exist dedicated transmembrane proteins, so-called membrane transporters, that enable the uptake of ions and polar molecules such as sugars and amino acids, enable cell communication and signaling, or mediate drug efflux leading to resistance against antibiotics in pathogenic micro-organisms, for example [1].

Currently, the substrates transported by many of these transporters are still unknown [2], and closing the sequence-annotation gap is an important task in bioinformatics [3]. Whereas certain transmembrane transport proteins can be assigned to their substrate class based on homology to a protein family, the exact substrate is often determined by a very small part of the sequence, while the remaining structure is an indicator of the transport mechanism, the protein family and the ancestry, respectively [4–6]. On top of that, large parts of known proteomes do not possess enough sequence similarity to related proteins to infer functional information from, and thus belong to the so-called *dark proteome* [7]. The ability to identify transporters for specific substrates within a (meta-)proteome is essential across various research fields, including biotechnology [8–10], pharmacology [11–13], and metabolomics [14, 15].

Machine learning has been successfully applied to many fields [16, 17], including a number of studies that aimed to assign substrate classes to membrane transporters. In 2010, Schaadt and Helms [18] trained Support Vector Machines (SVMs) on the frequencies of amino acid types in protein sequences, in order to distinguish between four substrate classes in *A. thaliana*. Other studies used sequence encodings based on multiple sequence alignments [19], position-specific scoring matrices (PSSMs) [20], and physicochemical properties of the amino acids in the sequence [21]. Text embedding methods adapted from Natural Language Processing (NLP) were also employed for protein annotation [3]. Previously [22], we introduced a machine learning approach that combined amino acid frequencies with different PSSMs for each protein, and optimized the feature dimensions through feature selection on the training dataset. This model achieved high evaluation scores on a dataset of transporters from *A. thaliana*, and performed even better when applied to a meta-organism dataset.

Machine learning classifiers achieve optimal performance when trained on homogeneous, well-separated classes, as this improves pattern recognition, reduces model complexity, and shortens training time [23]. However, the abundance of functional annotations and the complexity of their ontologies make it challenging to define classes of proteins that are distinct, while also providing useful information in their predictions [24]. In previous studies, classes for predictive models that aim to annotate substrates for all membrane transporters in a proteome were selected manually. Schaadt and Helms in 2010 [18], considered transporters belonging to four classes of substrates in *A. thaliana* (amino acids, oligopeptides, phosphates, and hexoses). Zhao and co-workers [21] split the transporters available in Uniprot (version 2013_03) into seven substrate classes: amino acid/oligopeptide, anion, cation, electron, protein/mRNA, sugar and *other*, and removed any transport protein annotated with more than one substrate. The TranCEP method [25] used this dataset for training as well. In our recent study [22], we considered four substrate classes (sugar, amino acids, potassium and electron).

However, relying on manually defined substrate classes has inherent limitations. Broad categories like *cation* may merge proteins with diverse sequences, structural folds, and transport mechanisms, potentially introducing confounding variables that prevent accurate predictions. Likewise, the choice of very specific substrate classes, such as *magnesium*, might not contain enough samples per class for training reliable machine learning models [22]. Selecting optimal substrate classes for each dataset requires extensive manual effort and biological knowledge. Therefore, we present an automated pipeline that generates distinct, homogeneous protein classes, using a greedy algorithm that optimizes class selection based on training data. Furthermore, the automated pipeline can rapidly compile convenient datasets for selecting substrate classes and training ML models across a variety of organisms, including bacteria, plants, mammals and yeast, and combinations of those.

Although methods for reducing redundancy in sets of GO terms were previously developed [24, 26], they were not optimized for machine learning tasks. Instead, these approaches were primarily designed for gene set enrichment analysis, operating on the entire GO and gene dataset. Therefore, they lack customization for specific datasets and tasks, such as the ability to identify annotations of transmembrane transporters that are both associated with a sufficient number of proteins for training and specific enough to provide meaningful information about the function of each transporter that is classified with the model.

With the recent rise of transformer models and natural language processing, first attempts have been made to adapt these methods to general protein function prediction [27]. Whereas most of these methods aim to assign annotations from the entire Gene Ontology (GO) [28] to the proteins, creating a small and non-redundant subset of functional annotations would considerably reduce the number of nodes in the output layer, leading to simpler models that, in practice, may provide a similar amount of information by their predictions.

Thus, the aim of this study was to implement a method that identifies an optimal set of transport-function-related GO terms, so that putative transporters can be reliably annotated with one or more of these terms. To this end, we implemented an algorithm that drastically reduces the number of GO terms a protein dataset is annotated with, and then returns a non-redundant subset with highly accurate pairwise classification results, that still contains the original functional information found in the complete set of GO annotations. Additionally, we introduce an improved software pipeline to compile protein datasets, along with functional annotations, that allows for filtering of transporter sequences by various measures, indicating data quality and other attributes. Unlike prior approaches that exclude multi-substrate proteins [18, 21, 22], our pipeline supports multi-label datasets, allowing a single protein to belong to multiple classes. This automated pipeline enables rapid and accurate identification of biologically relevant, separable transporter classes in a protein dataset, significantly reducing manual curation efforts.

## Materials and methods

### Data retrieval and preprocessing

Protein data was obtained from UniprotKB, version 2022_05 [29]. Proteins with fragmented sequences or without experimentally proven existence were not included in the dataset. After this initial filtering, a total of 1,693,707 proteins remained in the dataset. Non-standard amino acid codes, such as B, Z, J or X, were removed from the sequences. These amino acid descriptors were found in 45,718 protein sequences, although none of them were part of the membrane transporter datasets we explored in the results section.

The corresponding dataset 2022–11-03 of all Gene Ontology (GO) annotations [28], including those that were electronically inferred from other sources, was downloaded from the official GO webserver and filtered for those genes with Uniprot annotations. The relations between individual GO terms were downloaded from the Gene Ontology website, in the OBO format.

The code, along with links for downloading the raw data, is provided in the official repository (see Appendix 1).

### Dataset creation pipeline

A dataset pipeline was implemented that creates filtered protein datasets and annotates the proteins with a subset of GO terms. The protein sequences are first filtered for a set of organisms, while the GO terms can be filtered for those that are ancestors of a particular node (e.g. *transmembrane transporter activity*).

The proteins in the filtered Uniprot dataset are then annotated with the GO annotations from Section Data retrieval and preprocessing. Outdated GO terms are updated to their current version, using the *alt_id* node annotations in the GO graph. If a protein is associated with a particular GO term, then the ancestors of that GO term according to the filtered GO graph receive that relation as well.

For the transmembrane transporter dataset, only *molecular_function* GO terms that are descendants of *transmembrane transporter activity* (abbreviated as TTA) were kept in the dataset, and only edges with the *is_a* relation were allowed. Protein annotations were filtered for the *enables* qualifier, which denotes that the protein is directly responsible for the molecular function, as opposed to acting upstream, for example.

### Pairwise similarity scores

After creating a dataset of *n* proteins, and annotating them with a subset of *m* GO terms, we used the protein sequences of the annotated proteins, and other associated data, to create similarity matrices of size *m* × *m* between pairs of GO terms, with the goal of comparing these similarity scores to each other and to evaluation scores calculated from machine learning models.

#### Sequence similarity

In order to determine a “sequence similarity between two GO terms”, a matrix of pairwise sequence similarity scores between all proteins annotated with these two GO terms was computed. This matrix was then aggregated into a single number by taking the average, minimal or maximal value.

Pairwise sequence similarity scores were calculated with the Biostrings R-package, using the Needleman-Wunsch algorithm [30] for optimal global sequence alignments, with the BLOSUM62 substitution matrix [31] and gap opening- and gap extension penalties of 10 and 4, respectively.

#### Semantic similarity

Semantic similarity scores between pairs of GO terms were calculated using the GOSemSim R-package [32]. Here, we used the method proposed by Wang [33], as it can be calculated directly from the GO graph, and avoids the drawbacks of semamtic similarity measures that are calculated on term-based information content [33–35].

#### Overlap matrix

The overlap matrix for a set of *m* GO terms contains the number of proteins two GO terms have in common. Each position *i*, *j* < *m* in the matrix corresponds to the size of the intersection set between the set of proteins annotated with GO term *i*, and the set of proteins annotated with GO term *j*.

#### Evaluation of pairwise machine learning models

Machine learning (ML) models were trained to distinguish sets of protein sequences annotated with either one of two different GO terms.

To this aim, the protein sequences were transformed into numerical vectors of constant length by applying the feature generation algorithms described in [22]: Amino acid composition (AAC), pair amino acid composition (PAAC), and four PSSM features. The individual feature vectors were individually standardized along the sample axis, and concatenated into a single vector of length 1,600. The four PSSMs for each protein were calculated by calling PSIBLAST with different parameters: either one or three iterations of PSIBLAST, and either Uniref50 or Uniref90 [36] as the BLAST database, respectively. For this analysis, we did not cluster the dataset at a sequence identity threshold of 70% as we did before [22], since we wanted to estimate the correlation between sequence similarity and the performance of the machine learning models.

Class labels were derived from the GO annotation data described in Section Dataset creation pipeline. Depending on what type of model was used, all GO terms annotated to fewer than *k* or *k*_*u*_ proteins were removed from the dataset.

In our previous work [22], we observed that SVMs with RBF kernels were usually among the best-performing models, as long as the sample size was greater than 20, and no clear outliers could be found in the training dataset. While we previously removed multi-substrate transporters if both substrates were part of the classification task, we now used multi-output SVM classifiers that can be trained on a dataset where one sample can belong to multiple classes (e.g. when distinguishing proton transporters from proton+molecule symporters or antiporters). In the latter case, only pairs of GO terms were allowed where each term is associated with at least *k*_*u*_ ≤ *k* unique proteins in the dataset, excluding those that were part of the intersection. This was done to avoid cases where all or most proteins that are annotated with the first GO term are also annotated with the second GO term, which can, for example, occur when the former is the parent node of the latter.

Since the feature dataset consisted of 1,600 feature dimensions, but only hundreds or tens of samples, we added a feature selection algorithm to the machine learning pipeline. Based on the training dataset, the percentage *p* of best feature dimensions was selected, based on the ANOVA F-score between the respective feature dimension and the corresponding class label.

The performance of each model was evaluated by computing precision, recall and F1 scores individually for each class. The metrics represent the average score across five iterations of a nested cross validation, where the outer cross validation splits the data into a training dataset and an independent test set. An inner cross validation on the training dataset optimizes the hyper parameters of the model, that is then evaluated on the independent test set. The position *i*, *j* in the resulting matrix of pairwise evaluation metrics corresponds to the average F1 score on the independent test sets across all outer folds, when distinguishing the positive class *i* from the negative class *j*. The macro-averaged F1 score for a pair of GO terms was taken as the arithmetic mean between positions *i*, *j* and *j*, *i*.

### Greedy algorithm for clustering of GO terms

GO terms can be closely related in terms of function, and therefore have many annotated proteins in common. Trying to distinguish two very similar GO terms from each other through means of machine learning can be a challenge, since the small number of unique samples available for each class makes it harder for the ML model to find the correct patterns in the feature vectors, and since the function may be too similar. For that reason, we implemented a pipeline that reduces the redundancy in a subset of GO terms.

First, all GO terms with fewer than *n* associated proteins were removed from the dataset, since they would anyhow not possess enough samples available for training a ML model. In some cases, we also removed the top *p*th percentile of GO terms according to the distribution of sample counts. For the remaining GO terms, a sparse matrix of pairwise ML evaluation scores was created. For each pair of GO terms, a multi-output SVM with ANOVA-based feature selection was evaluated by a 5-fold cross-validation approach. The average F1 score across the respective test sets of CV iterations was then used as a measure for how well the model can assign the correct GO terms to the associated protein sets, using the ML features described in Section Evaluation of pairwise machine learning models. Evaluation scores were only calculated for pairs of GO terms where both have at least *m* unique proteins that are not annotated with the respective other GO term. If not enough unique proteins were available for training a classifier on a pair of GO terms, the respective position in the matrix was left empty. This threshold *m* was necessary to avoid cases where the annotated proteins of one GO term are a subset of the annotated proteins of another GO term, which would leave us with little or no training data for the minority class.

The set of GO terms was then optimized via a greedy algorithm that iteratively removed one GO term per step. For each GO term *g* in the subset, the coverage of remaining GO terms (i.e. those that have not been removed yet and are not equal to *g*) was calculated by dividing the number of proteins annotated with those GO terms by the total number of proteins. If removing a particular GO term would not cause the coverage to fall below a specified threshold, that GO term was considered as a candidate for removal. For each of these GO terms, the potential impact (increase) of its removal on the average F1 score was calculated. When calculating the average, the empty positions in the F1 matrix were filled with a specified constant value, by default with -1, which ensures that these GO terms get lower average scores and are removed first. The list of GO terms considered for removal was then shortened to those with the highest positive impact on the average F1 score when deleted, plus a small term *ϵ*, which causes GO terms with a very similar impact on average F1 scores, e.g. a difference of less than 0.001, to be considered to have the same impact.

The GO terms with the highest positive impact were sorted by their level in the GO graph (i.e. the distance from the selected root node), and either the most abstract or the most concrete GO term was selected for removal, according to the preference of the user. Should there still be multiple GO terms on the same level, one of those terms was drawn by a deterministic random number generator and removed from the set of GO terms. This method was repeated, until no more GO terms could be removed without the coverage falling below the specified threshold.

The exact algorithm used for optimizing GO annotation sets is described in more detail in Appendix 4.

### Pipeline overview and complexity

The full pipeline, including transporter dataset creation, feature calculation, pairwise machine learning model evaluation, and application of the greedy algorithm, is outlined in a flowchart available in S11 Fig. The entire software package makes extensive use of caching, so after an initial pre-calculation phase that requires up to a few hours on a modern desktop processor, the entire pipeline can subsequently be executed within seconds. Pre-calculated PSSMs for the feature calculation are provided together with the raw data, as the calculation of four PSSMs for each protein is the most time-intensive task. During the pre-calculation step for the greedy algorithm (see Section Evaluation of pairwise machine learning models), a total of $\frac{k^{2}-k}{2}-j$ ML pipelines are trained and evaluated, where *k* is the initial number of GO terms and *j* is the number of GO term pairs that share too many proteins to have enough samples per class for training (e.g. when two annotations are parent and child nodes in the Gene Ontology graph). The greedy algorithm itself removes GO terms until a number of conditions are met, meaning that its linear runtime has an upper bound of *k*.

## Results

### Dataset creation pipeline

First, we employed an updated version of the transporter dataset creation pipeline described in [22] to create a meta-organism transmembrane transporter dataset. This dataset included transporters from four organisms: *Arabidopsis thaliana, Saccharomyces cerevisiae, Escherichia coli* and human.

The proteins were filtered according to multiple criteria that measure the quality of the underlying data: The manual review status by Swissprot reviewers, whether there are known genes associated with the proteins, the type of evidence used to annotate the protein with a particular GO term, and whether the protein is still part of the dataset after applying sequence clustering with a particular threshold (see S1 and S2 Tables) shows the number of transport-related GO terms that these proteins are annotated with.

When selecting only manually curated data based on experiments, we obtained a total of 1.602 transporters at a clustering threshold of 70%. With all of Uniprot and the same parameters, we would only get additional 250 proteins, meaning that these four organisms provide an adequate representation of all organisms in Uniprot. The transporters in this meta-organism dataset were annotated with 679 transmembrane-transporter-related GO terms, compared to 639 for all of Uniprot. This was due to the sequence clustering, where lower clustering thresholds are more likely to assign two proteins with different GO terms into the same cluster, thereby potentially removing the GO terms of one of these proteins from the dataset.

### Application of the redundancy reduction pipeline to the meta-organism dataset

**Fig 1:**
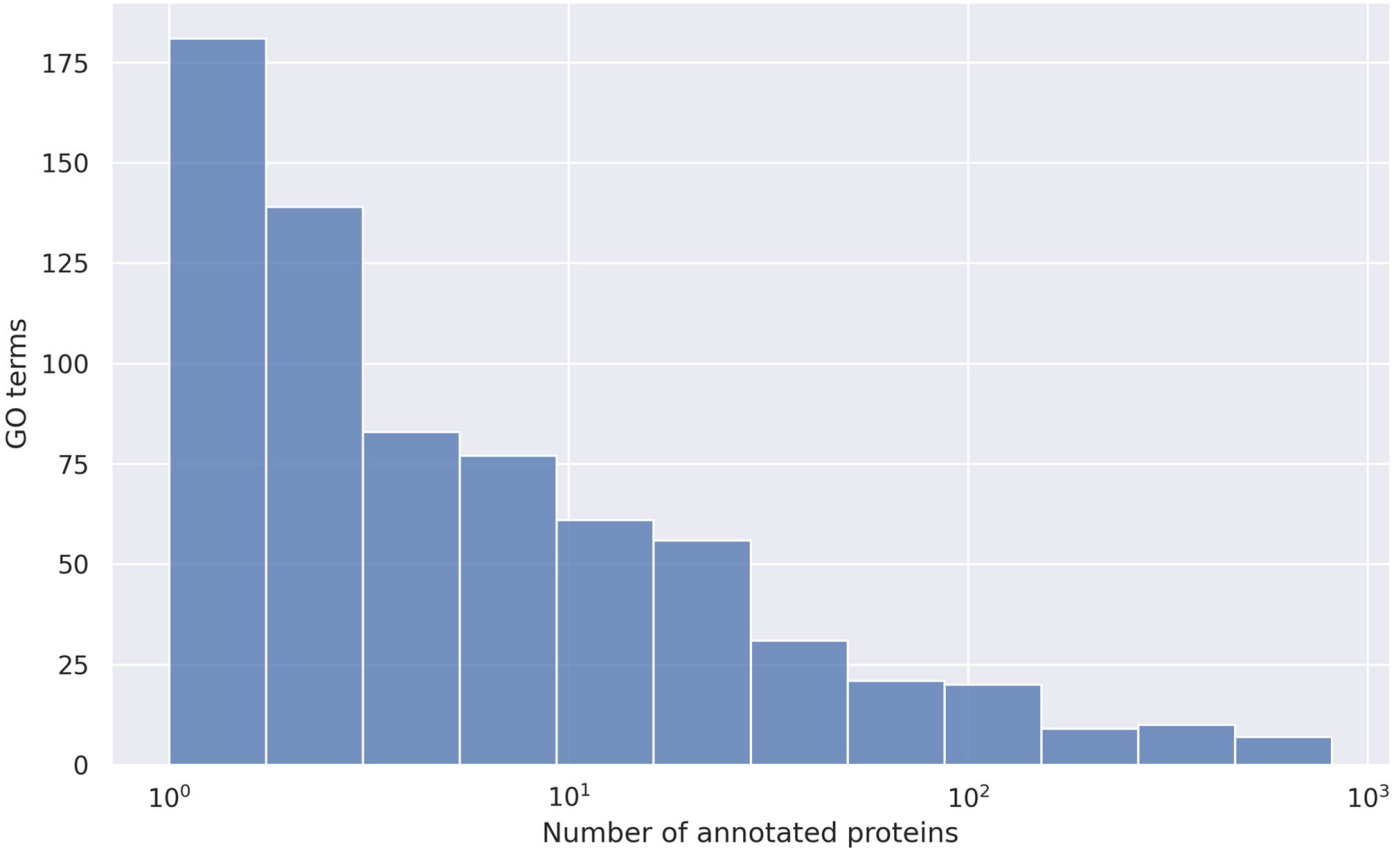
Distribution of annotated protein count for membrane transporter GO terms. Number of annotated proteins for molecular function GO terms related to transmembrane substrate transport. The dataset only contains SwissProt proteins and GO term annotations based on experimental data.

Next, the GO term clustering pipeline described in Section Greedy algorithm for clustering of GO terms was applied to this dataset. Due to the large number of proteins, we found it necessary to not only remove GO terms with fewer than 20 proteins from the dataset, but to also remove the top *p*th percentile of most general GO terms, according to the distribution of protein counts. Protein counts follow a steep exponential distribution, with a median of 4.0 and a maximum of 815, excluding the root node *transmembrane transporter activity* (see Fig 1). When keeping GO terms at or below the 99th percentile, which lies at 454.44, only seven out of 695 GO terms are removed. Removing the 35 terms above the 95th percentile at 110.6 leaves us with 660 GO terms.

Before applying the greedy algorithm and when calculating pairwise F1 scores with *m* = 10 and after removing GO terms with less than *n* = 20 proteins, the dataset consisted of 1661 proteins annotated with 137 GO terms. For 1.666 pairs of these GO terms, no evaluation scores were available since their protein sets were too similar to train a ML algorithm on them. The median F1 score for the remaining pairs was 0.916.

With *p* = 0 (i.e. when not removing the top *p*-th percentile GO terms according to the number of annotated proteins), the median F1 score at a coverage of 100% starts at around 0.845 (see S2 Fig). Subset sizes are comparatively small (see S3 Fig), many pairs without F1 scores still remain in the dataset (see S4 Fig). This is doe to the presence of abstract GO terms that cover many proteins, but also have large overlaps with each other, such as *channel activity* or *secondary active transmembrane transporter activity* (see S10 Fig). After the greedy algorithm removed these abstract terms, they were replaced by their child terms, which are more precise and have less overlap. This more than doubled the number of GO terms needed to reach the required coverage, and caused the number of pairs without scores to drop, while the median score rose sharply. At lower coverage thresholds, the subset sizes dropped again, while the F1 scores and available pairs continued to improve.

**Fig 2:**
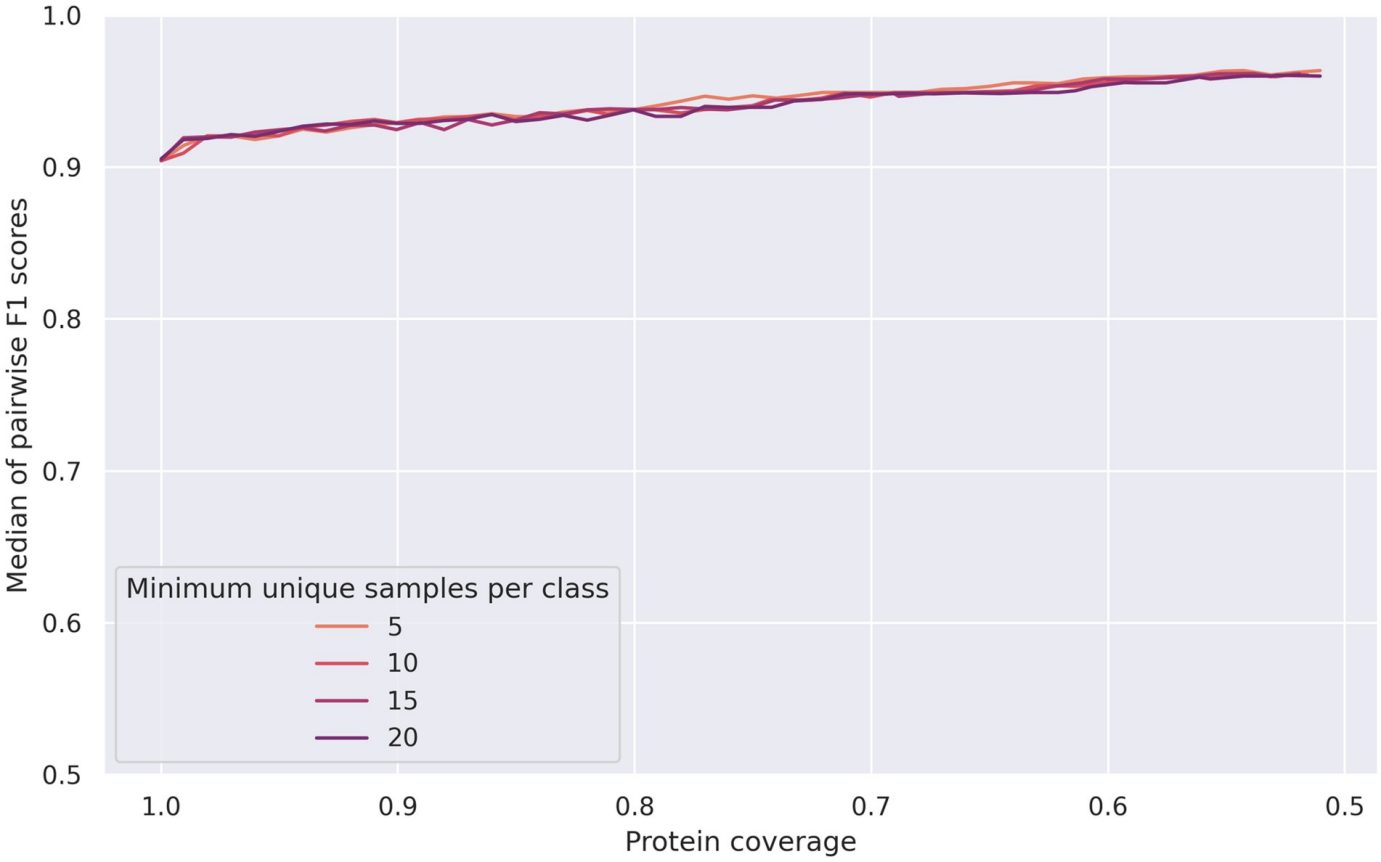
Median F1 scores for meta-organism dataset at different coverage thresholds, when removing most abstract terms. Median F1 scores between pairs of GO terms for the meta-organism dataset, after removing the top 5th percentile of GO terms according to sample count.

**Fig 3:**
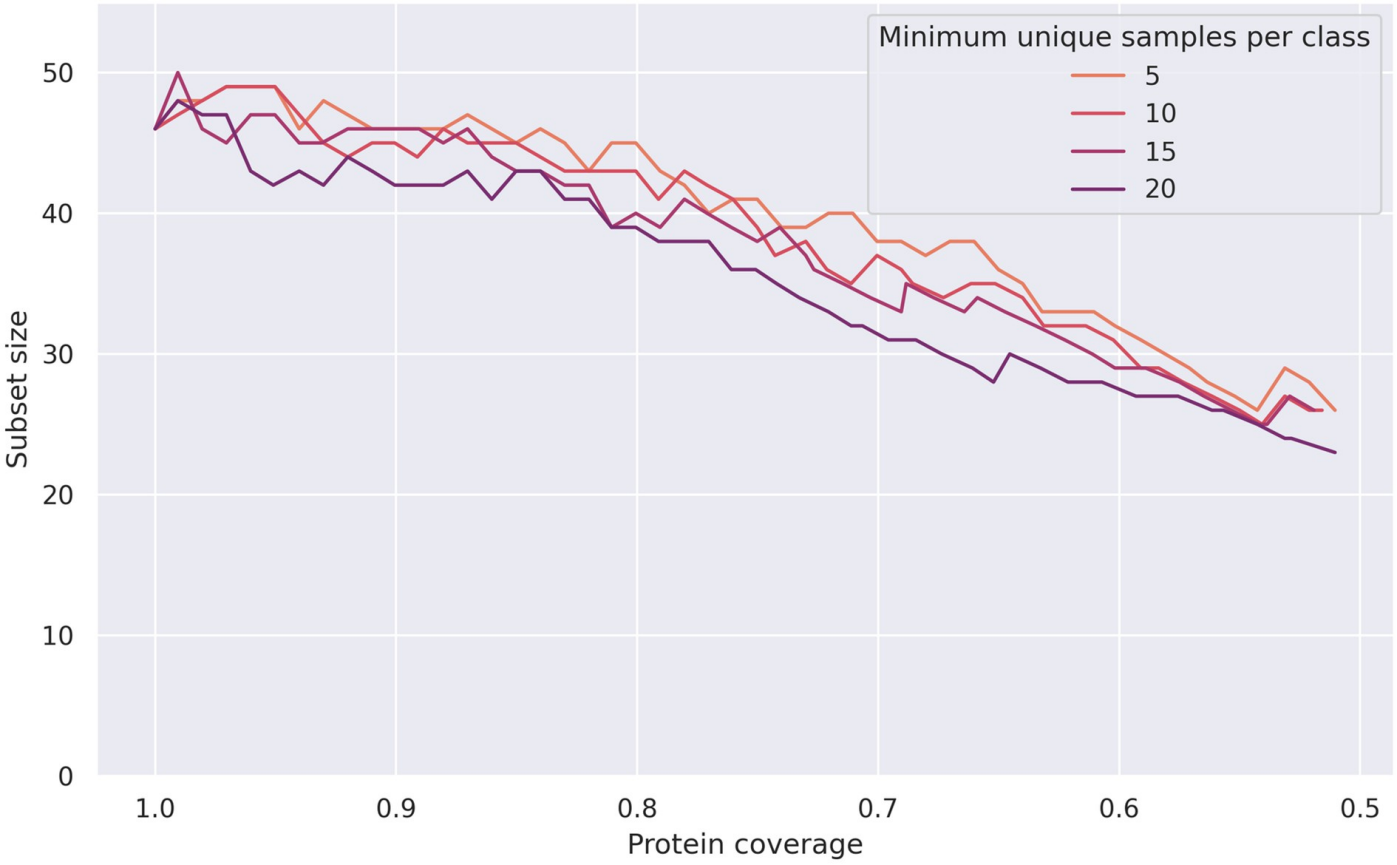
Subset sizes for meta-organism dataset at different coverage thresholds, when removing most abstract terms. Final subset sizes for the meta-organism dataset generated by the pipeline, after removing the top 5th percentile of GO terms according to sample count.

**Fig 4:**
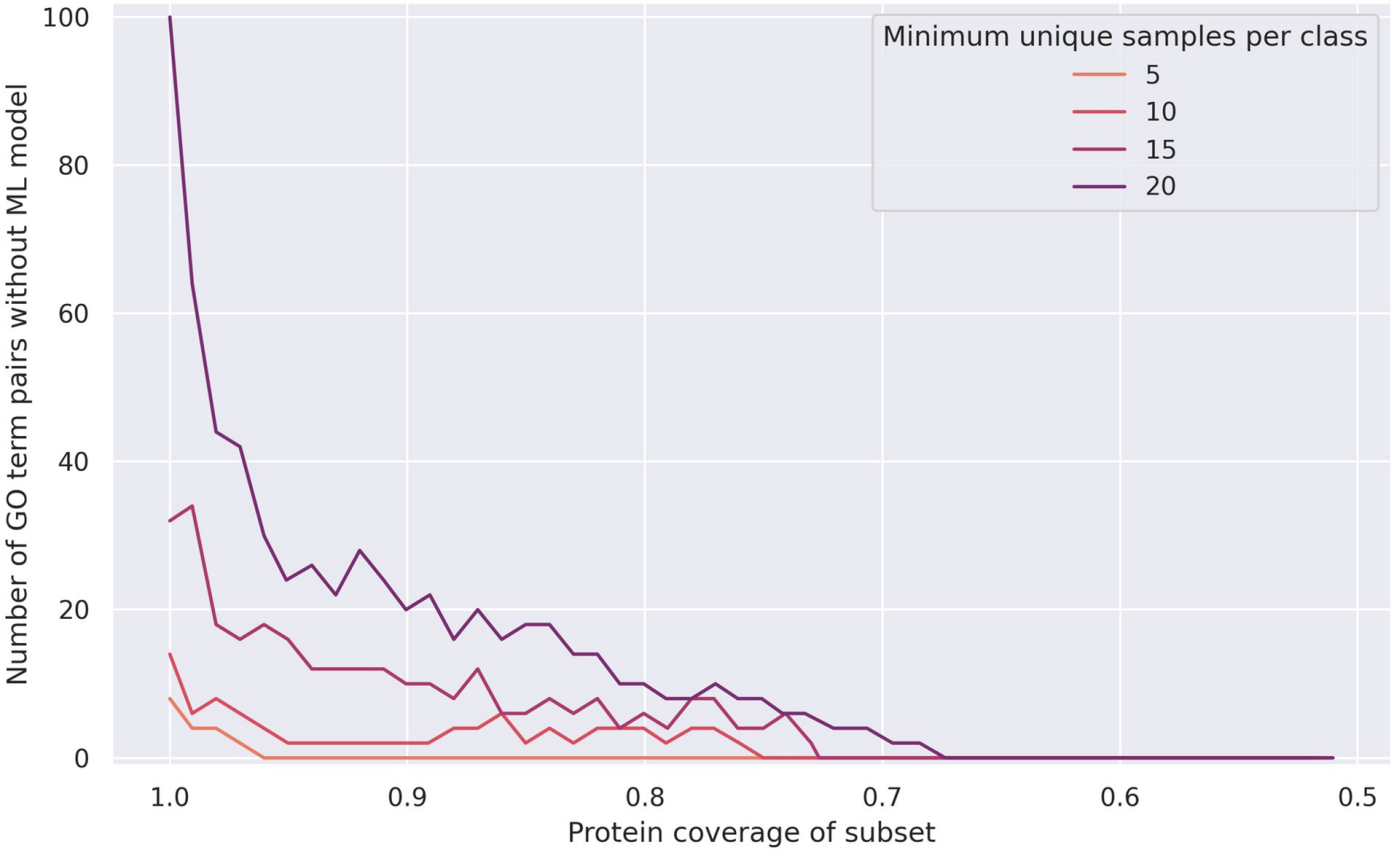
ML models excluded due to low sample count for the meta-organism dataset, when removing the most abstract terms. Number of GO term pairs without F1 scores for the meta-organism dataset due to a unique sample count of less than *m*, after removing the top 5th percentile of GO terms according to the sample count distribution.

**Fig 5:**
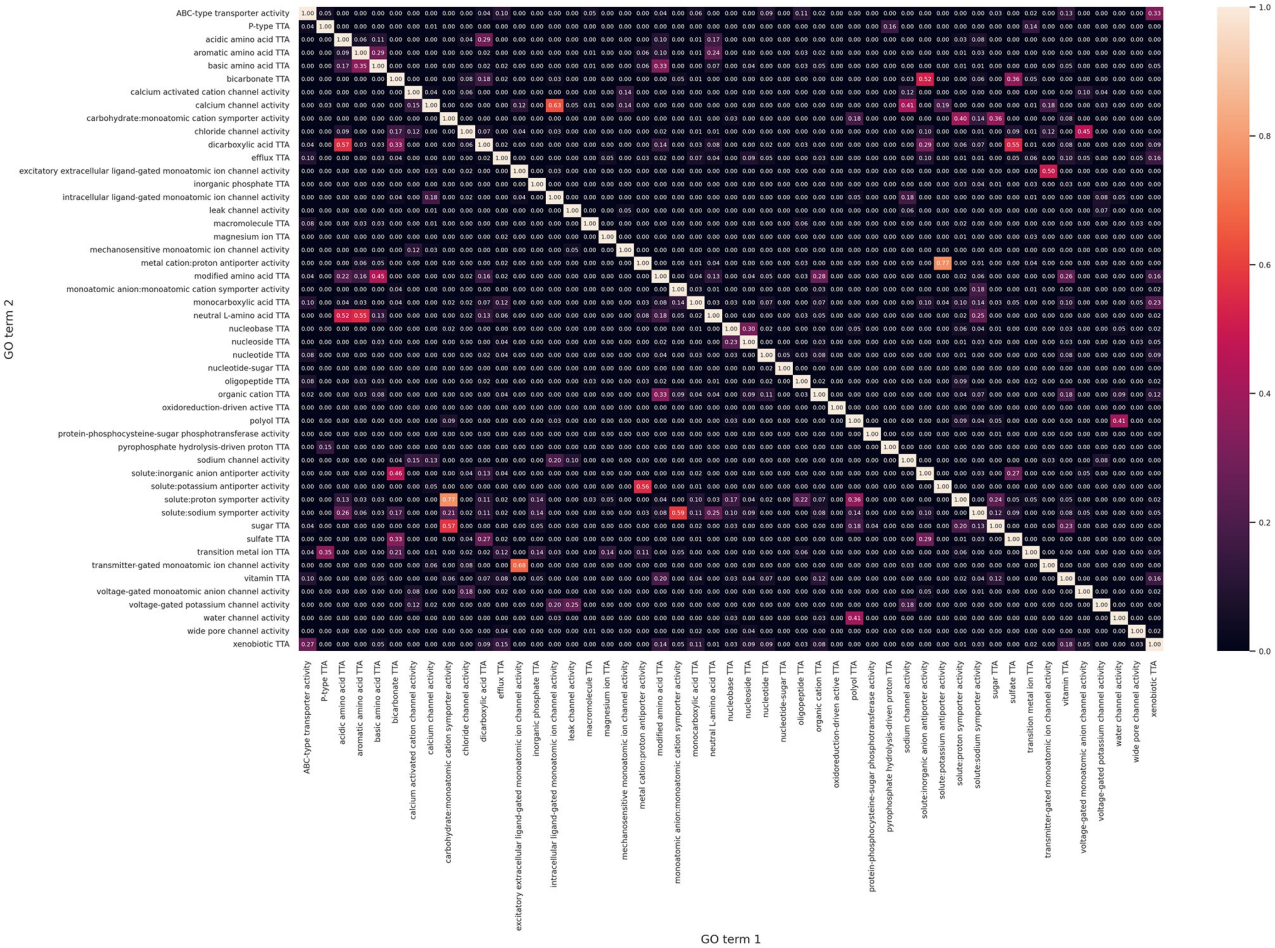
Overlap heatmap for all transporters in meta-organism dataset. Heatmap showing the fraction of proteins annotated with GO term 1 that are also annotated with GO term 2, for all pairs of GO terms in the optimized subset for the meta-organism dataset.

Removing the top 5th percentile of GO terms at the beginning of the pipeline with *p* = 5 means that they do not have have to be removed by the greedy method, and already yields good results at a coverage threshold of 100% (see Figs 2, 3 and 4). With *m* = 5 and a coverage threshold of 0.96, which was the highest coverage for which there were F1 scores available for all pairs, we found a subset of 49 GO terms, with a median pairwise F1 score of 0.92±0.10. Overlaps between the terms were low, the mean overlap was 2% and the maximum overlap was 77% (see Fig 5).

Finally, we calculated the best subsets with *m* = 20. At this number of unique proteins per GO term, the highest possible coverage is 67% with *p* = 5, and 60% with *p* = 0 (see Fig 4, S4 Fig). The resulting subset of length 30 reached a median F1 score of 0.95±0.06. Overlaps were minimal (see S8 Fig), with a mean and maximum overlap of 1% and 42%, respectively. In conclusion, setting *m* to 20 represents a trade-off between coverage and subset size. After the lowest possible redundancy is reached (as in Fig 5), the number of GO terms can be further reduced by lowering the coverage threshold until pairwise F1 scores are available for all GO terms at *m* = 20. This also has a positive impact on the pairwise F1 scores.

### Comparison of meta-organism results with substrate classes used in previous studies

Finally, we compared the results of this study to previous machine learning works that classified a set of transmembrane transporters into substrate classes. For this, we searched the meta-organism dataset for GO terms that matched the corresponding substrate transport functions. We evaluated the resulting sets of GO annotations in terms of protein coverage, pairwise F1 scores, and annotation overlap. Previous studies used keyword annotations from UniprotKB to retrieve substrate classes, and one of these substrates was *electron*. However, in the Gene Ontology, which was used in this study as basis of functional annotations, *electron transfer activity* is classified as a molecular function that is separate from *transmembrane transporter activity*, rather than a descendant of it. Therefore, membrane proteins that enable reactions of electron donors and electron acceptors were not part of the new dataset, and were not available for comparison.

In 2010, Schaadt and Helms only considered four substrate classes of *phosphate*, *amino acid*, *oligopeptide*, and *hexose* [18]. In our dataset, using that subset of proteins would have only covered 17.8% of transporters. The overlap between the classes was small, only *amino acid* and *oligopeptide* had an overlap of 6%. The average pairwise F1 score was 0.83±0.14.

In 2014, Mishra et al. [21] also considered a category of *other transporters* based on data from version 2013_03 of Uniprot. However, it was unclear to us which substrates were included in that category. Furthermore, electron transfer proteins were not part of our dataset. The remaining five classes of *sugar*, *protein*, *cation*, *anion* and *amino acid* provided a protein coverage of 66.6%, with average F1 scores of 0.85±0.13. Three substrate classes (amino acid, anion and sugar) had overlaps of 22–36% with the cation class, in part because cation symporters and antiporters were included in the dataset. Otherwise, the overlaps were small.

Finally, we analyzed the substrate classes that we used in our previous study [22]. When excluding the electron transfer proteins, the remaining three substrate classes *sugar*, *amino acid* and *potassium* had no pairwise overlaps and covered 26.2% of the meta-organism dataset, with average pairwise F1 scores of 0.9±0.05.

By comparison, the meta-organism GO subset of 49 GO terms that was optimized for high protein coverage included 96% of transporters, with median F1 scores of 0.92±0.1. The median overlap was 0.0, with a small number of outliers that were necessary to reach the high coverage (see Fig 5). When accepting a lower coverage of 67%, we found a subset of 30 GO terms with median F1 scores of 0.95±0.06, and much smaller overlap between the classes (see S8 Fig), meaning that the increased detail of the larger subset led to better classification performance and more consistent ML models.

### Yeast transporter dataset

#### Results of the dataset creation pipeline

In a second stage, we analyzed a single-organism dataset, containing transporters from *S. cerevisiae*. Here, all transporters in our dataset are part of the manually curated SwissProt database, meaning that considering data from TrEMBL would not have increased the sample count. The gene names of all proteins in the dataset were known. The final protein dataset contained 332 transport proteins. Sequence clustering with CD-HIT [37] at a similarity threshold of 70% would reduce that number to 303.

When applying the annotation pipeline described in Section Dataset creation pipeline, the transporters were directly annotated with 211 unique transport-related molecular function GO terms. Adding the ancestors of these GO-MF terms to the dataset increased that number to 288. Overall, our yeast dataset contained a total of 3,299 unique relations between transport proteins and transport-related functional GO terms, 380 of which were inferred by electronic annotation (IEA).

#### Evaluation score matrix from machine learning models

**Fig 6:**
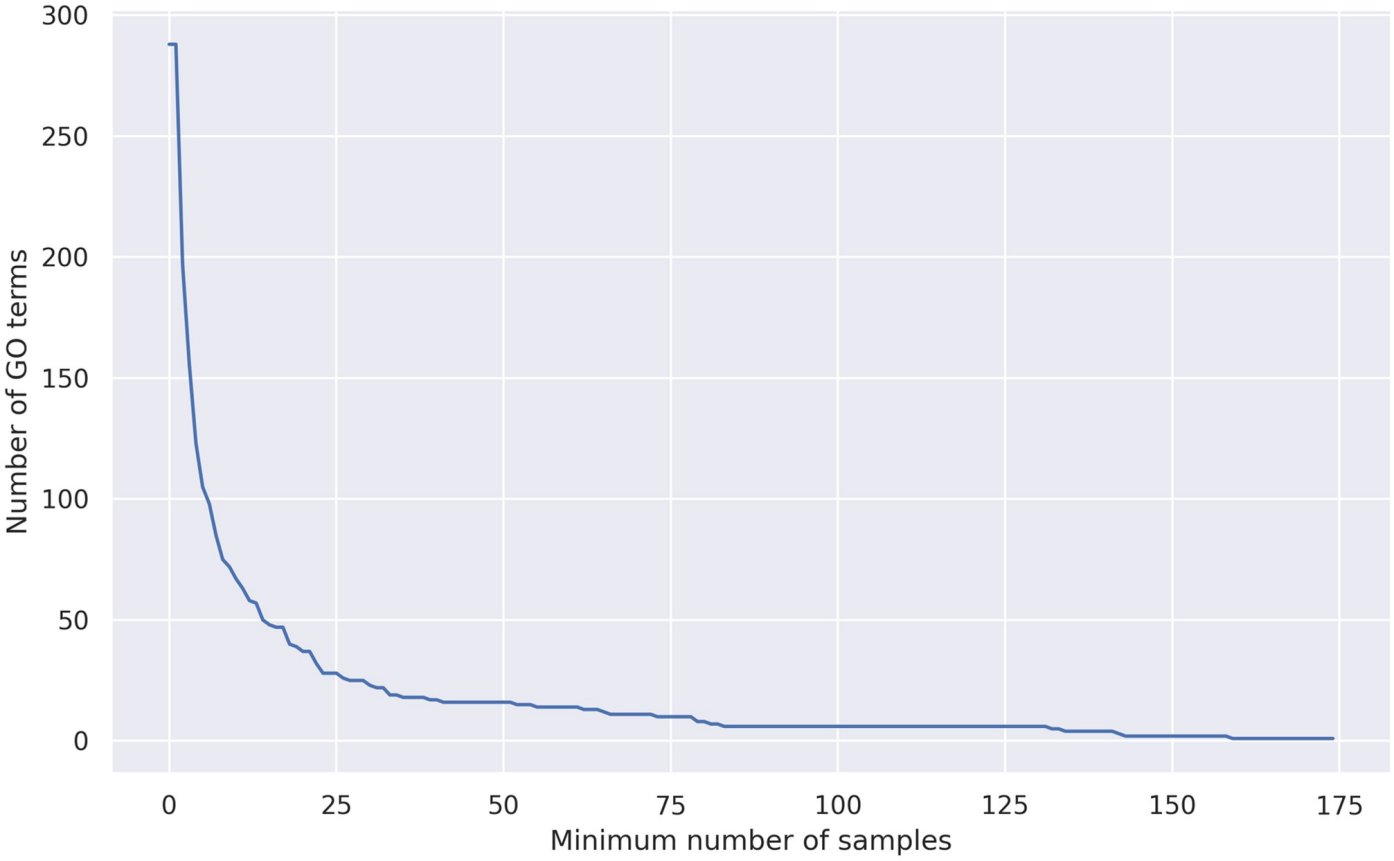
Number of GO terms associated with more than a specified number of proteins in yeast. Number of GO terms available for training, at different thresholds for the minimum number of proteins associated with any GO term in the yeast dataset.

Previously [22], we showed that at least 20 proteins per class are necessary to predict substrates of transmembrane transporters with good accuracy. Therefore, we pruned the GO terms in our dataset to those with at least 20 associated proteins. This represents a tradeoff, because the number of GO terms available for classification decreases rapidly if the minimum required number of proteins per GO term is raised (see Fig 6). Filtering the GO terms in this way did not decrease the number of proteins in our dataset, since they were still annotated with the ancestors of these more specific terms.

Next, we generated a sparse matrix containing the evaluation scores of pairwise machine learning classifiers, as described in Section Evaluation of pairwise machine learning models. Each matrix entry is equal to the score of a multi-output SVM-RBF classifier that annotates a protein sequence with either one or both of the GO terms. Models were only trained on pairs of GO terms where each term was associated with *m* unique proteins, i.e. those that were not in the intersection set between the protein sets of the two GO terms. The final matrix had 37 dimensions. With *m* = 20, 524 out of 1,332 distinct pairs of GO terms were not suitable for evaluation due to a lack of unique samples in at least one of the classes.

After performing a nested cross validation for each pair of GO terms, the median F1 score on the independent test sets was 0.91 (see S3 Table). In order to test the impact of feature selection and dimensionality reduction on the scores, we trained two additional ML pipelines that employed either ANOVA-based feature selection or PCA to reduce the number of feature dimensions, based on the respective training dataset at hand. While feature selection did not substantially impact the median F1 score, it improved the minimum scores between pairs of GO terms from 0.15 to 0.23 and 0.37 for PCA and ANOVA, respectively. Overall, the model with ANOVA-based feature selection seemed to perform best.

Finally, we repeated the same tests after removing redundant sequences with more than 70% sequence similarity from the dataset using cd-hit. This reduced the number of proteins in the dataset from 332 to 303, and the number of GO terms with more than 20 associated proteins from 37 to 32. Median F1 scores on independent test sets between all GO term pairs were between 0.02 and 0.04 lower across the different models (see S3 Table).

#### Comparative analysis

Now we wanted to find out which pairs of GO terms can be well distinguished and which ones not. Intuitively, one may expect that it is more difficult to distinguish transporters of substrates A and B when either the protein sequences are highly similar to each other or when A and B are very similar. To this aim, we compared the model evaluation scores of binary machine learning models trained on pairs of GO terms to similarity scores for those GO terms.

The data was split into two subsets: GO term pairs with a macro-averaged F1 score below 0.75, and GO term pairs with an F1 score above or equal to 0.75. This threshold was selected based on the minimum sample size in each set, where a lower value would result in fewer than 30 GO term pairs in the subset of low-performing pairs, which could lead to biased results.

We tested using the Shapiro-Wilk test [38] which of the similarity scores follow a normal distribution. The median and mean sequence identities, and the GO semantic similarity had a test statistic higher than 0.9, indicating they follow a normal distribution.

For each of these normally distributed variables, we performed a one-tailed independent t-test between the two subsets of low-performing and high-performing GO term pairs, with the alternative hypothesis that the mean similarity score of the pairs with low F1 score was smaller than the mean of the population. After multiple-hypothesis correction by the Benjamini-Hochberg method, only the mean sequence identity (p = 0.0306) was statistically significant with *p* < 0.05. No statistically significant similarity scores were found for the other tail of the distribution.

For the similarity scores that did not follow a normal distribution, we performed two non-parametric Mann-Whitney-U tests between the two subsets for each score, one for each tail of the distribution. After Benjamini-Hochberg correction, the mean sequence identity and the minimal sequence identity had statistically significant p-values, with the alternative hypothesis that pairs with lower F1 scores have a lower similarity score. For the other alternative hypothesis, i.e. that pairs with lower F1 score have a higher similarity score, we found the absolute difference in sample count, the maximum sequence identity between proteins annotated with the GO terms, and the semantic similarity to be statistically significant.

In summary, good ML separability between a pair of GO terms requires (unexpectedly) that the sequence similarity between the associated proteins should be higher than a certain minimum, but also (expectedly) should not exceed a certain maximum. The Wang method [33] for calculating semantic similarity between GO terms was also able to predict good ML performance, meaning that proteins annotated with more dissimilar molecular functions are also better separable by the ML model.

In addition, we also calculated correlation coefficients between F1 scores and similarity measures of all pairs in the dataset. Spearmans rank correlation was used, since most of the tested variables were not normally distributed. The F1 scores on the training and test data showed a very high correlation of 0.956, meaning that the trained models adapt well to the independent test sets. The highest correlation (0.44) was observed for the minimum sequence identity, the lowest correlation (-0.55) for the maximum sequence identity (see Table 1), which corresponds to the results obtained from the statistical tests.

**Table 1:** Spearman correlation between F1 scores and similarity measures.

score	mean train score	mean test score
go max sequence identity	-0.602	-0.555
overlap	-0.475	-0.421
sample count diff	-0.446	-0.420
go semantic similarity	-0.331	-0.312
go median sequence identity	-0.054	-0.032
go mean sequence identity	-0.039	-0.017
go min sequence identity	0.442	0.405
mean train score	1.000	0.957
mean test score	0.957	1.000

Finally, we calculated mean F1 scores of individual GO terms across the pairs and compared them to a variety of attributes, including the number of annotated proteins and the level (i.e. the distance from the root node). The highest positive correlation with the test F1 score was found with the level (0.39), while the lowest (-0.61) was with the number of annotated proteins. This means that class labels should not be too abstract, since the set of protein sequences annotated with very broad GO terms is more diverse, making it harder for the machine learning model to find patterns.

#### Applying the GO term clustering algorithm to the yeast dataset

After removing all GO terms with fewer than *n* = 20 samples from the *S. cerevisiae* dataset, we were left with 36 out of 287 transmembrane transport-related GO terms.

Since the multi-output classifiers we used are capable of annotating proteins with one or both of the GO terms, we needed to filter out pairs where the minimum number of unique proteins per class, i.e. proteins that were not part of the intersection of the classes, was higher than a threshold *m*. We tested four different values for the parameter *m*: 5, 10, 15 and 20. Setting *m* to a value lower than five was technically not possible in our workflow, since we need at least five unique proteins for the 5-fold cross validation. Increasing *m* leads to a smaller number of pairs with F1 scores available in the pairwise evaluation matrix at high coverage values (see S5 Fig), but leads to more consistent median F1 scores (see S6 Fig). Based on these findings, we selected *m* = 10 as a trade-off between high coverage of the resulting subset and large enough sample count during evaluation of the pairwise SVM models. Before optimizing the subset, evaluation scores were available for 244 out of 1296 possible pairs, and the median F1 score was 0.90. Applying the greedy optimization algorithm described in Section Greedy algorithm for clustering of GO terms with *m* = 10, *ϵ* = 0 and a preference for less abstract terms yielded a subset of 11 GO terms that cover 98.1% of all transmembrane transport related proteins in *S. cerevisiae*. F1 scores were available for all pairs of GO terms in the optimized subset, the median score was 0.87 (see Table 2). The pairwise overlap matrix of the subset (see S1 Fig) has a relatively low median value of 0.07 (arithmetic mean of 0.12), with a maximum pairwise overlap of 0.66. This is mostly due to the fact that abstract terms such as *primary active transmembrane transporter activity* need to remain in the dataset in order to reach the 98% coverage, since many of their child terms do not have enough annotated proteins to be included. For the meta-organism dataset, this issue was overcome when we included proteins from other organisms. Asking the pipeline to prefer more abstract terms did not have an effect on the subset that was found by the algorithm, with the given parameters.

**Table 2:** Subset of GO terms selected for the yeast dataset when prioritizing more specific terms and optimizing for protein coverage.

GO term	Proteins
amide TTA	21
carbohydrate derivative TTA	25
carboxylic acid TTA	64
monoatomic cation TTA	131
organophosphate ester TTA	22
passive TTA	32
primary active TTA	61
protein TTA	21
salt TTA	72
secondary active TTA	80
sulfur compound TTA	21

Next, we tried other values of *ϵ*, along with ten different random seeds for the random number generator. Higher values of *ϵ* cause more GO terms to be considered to have the same impact on the average F1 score, therefore making the results more dependent on the random draws that happen at the end of the pipeline. This can be used as an additional optimization step, in cases where the greedy algorithm finds a local minimum. Results were filtered for those subsets with high coverage (98% or more) and with F1 scores available for each pair. With *m* = 10, all resulting subsets were made up of 11 GO terms and a minimal variance between the results obtained with different random seeds and *ϵ* values. When telling the algorithm to prefer abstract terms and setting *ϵ* to 0.005, a subset with a slightly higher coverage was found (99% compared to the previous 98.1%), with a median F1 score of 0.87 (see Table 3). The resulting subset showed a higher average and median overlap (0.16 and 0.1, respectively), and a much larger maximum overlap of 0.91. While preferring abstract terms can increase the protein coverage, for example by selecting *organic acid* (65 proteins) instead of its child term *carboxylic acid* (64 proteins), the more generalized terms are more likely to annotate other proteins in the dataset, leading to larger pairwise overlaps.

**Table 3:** Subset of GO terms selected when prioritizing abstract terms and optimizing for protein coverage, on the yeast dataset.

GO term	Proteins
amide TTA	21
carbohydrate derivative TTA	25
inorganic molecular entity TTA	158
macromolecule TTA	21
monoatomic ion TTA	142
organic acid TTA	65
organophosphate ester TTA	22
primary active TTA	61
salt TTA	72
secondary active TTA	80
sulfur compound TTA	21

## Discussion

Here, we aimed at identifying an optimal set of molecular function GO terms to split the members of a membrane transporter dataset, where optimality is meant in terms of separability. As an analogy, one could try to classify humans into those that like pop music, jazz, classical music or none of all. One question would then be whether it is beneficial to subdivide classical music into sub-categories, or to remain at the more abstract level of classical music. The analogy of music styles are GO terms at different abstraction levels of the Gene Ontology.

First, we implemented a software pipeline that automatically generates datasets of specified transmembrane transporter proteins, where the proteins and their functional annotations can be filtered for a variety of attributes, such as data quality, organism or the cellular compartment where they carry out their functions. Using this pipeline we created two transmembrane transporter datasets: A single-organism dataset containing only proteins of *S. cerevisiae*, as well as a meta-organism combining the transporters of human, *S. cerevisiae*, *E. coli* and *A. thaliana*.

Then we implemented an algorithm for reducing the redundancy in a set of GO terms, in order to find an optimal set of classes for training a machine learning algorithm. This greedy algorithm aims to minimize the pairwise overlaps of annotated protein sets, while producing subsets containing either abstract terms or specific terms. A filter for minimum and maximum protein count of the GO terms is employed, in order to reduce major differences in sample size.

When applying the pipeline to the meta-organism transporter dataset, the optimized subset contained 49 out of 695 functional annotations, which provided a coverage of 96%, a median pairwise F1 score of 0.92±0.10, and a mean overlap of 2%. Lowering the minimum required protein coverage to 67% reduced the subset size to 30 GO terms, with a median F1 score of 0.95±0.06 and a mean annotation overlap of 1%. This was an improvement on previous attempts at dividing transporters into substrate classes, which either provided a much smaller protein coverage, or led to lower pairwise F1 scores because of broader functional annotations. For the yeast dataset, the algorithm lowered the number of transporter GO terms from 287 to 11, with a median pairwise F1 score of 0.87±0.16 and covering 98.1% of transmembrane transporters.

Also, we tested for the yeast dataset what characteristics of two sample groups determine whether they are well separable or not. To this end, we computed multiple pairwise similarity measures for the GO terms. On each pair of GO terms, we trained and evaluated a multi-output SVM model with RBF-kernel, using ML-features derived from the amino acid sequence and from evolutionary information. Model evaluation was carried out in a nested 5-fold cross validation approach, and the average F1 score across the five test sets was used as a metric for how well the model can distinguish proteins of these two classes. When only evaluating pairs of GO terms where each GO term has at least *m* = 20 unique samples available for training, the SVM-RBF model with our combined ML-feature and ANOVA-based feature selection achieved a mean F1 score of 0.88±0.11 on the test sets for the *S. cerevisiae* dataset (see S3 Table).

Then, we compared the similarity scores to these F1 scores on the yeast dataset using correlation coefficients and statistical tests. We found that the strongest predictor of low F1 scores was a low mean sequence identity between proteins annotated to the two different GO terms. On the other hand, both a lower minimum sequence identity and a higher maximum sequence identity between pairs of proteins annotated with the GO terms are predictors of low F1 scores. Together, this means that the protein sets should not be too similar, but also not too dissimilar to each other. Another predictor of a low evaluation score was a large difference in sample count, so comparing very abstract to very specific functional annotations can lead to poor results. The GO semantic similarity was also able to predict good separability with machine learning models.

While correlations with machine learning performance were observed, such as with minimum and maximum sequence identity (see Table 1), these correlations were not strong enough to make decisions about which annotations to keep and remove. Hence, we implemented a greedy algorithm that optimizes class selection based on the training dataset. Although the requirement of at least 20 samples per functional annotation prevents the inclusion of very specific GO terms, we addressed this limitation by adding the ability to create meta-organism training datasets that can include proteins from plants, bacteria, single-cell eukaryotes, and mammals. Multi-output machine learning algorithms, which allow training on proteins with multiple substrates are helpful in increasing the number of available samples per annotation.

Our method offers an automated solution to identify which functional annotations of transmembrane transporters can be separated with sufficient accuracy through machine learning. It provides a pipeline that vastly reduces the redundancy in a set of functional annotations, using a greedy algorithm and training data. Another pipeline allows for the creation of custom training datasets for a variety of organisms, including plants, single-cell eukaryotes, bacteria and mammals. This approach reduces the need for extensive manual effort in simplifying datasets, and helps in improving machine learning model efficiency, classification performance, and training time. This brings us closer to the goal of creating predictive models that can automatically annotate entire proteomes and metaproteomes with transmembrane transport annotations and transported substrates, providing valuable information for various fields of research [8, 9, 13, 14].

## Appendix 1

### Code and data

The code developed for this study was uploaded to GitHub as a Python package, along with a link to download the raw data, and Jupyter Notebooks that reproduce the results. The repository can be found under https://github.com/adenger/subpred4.

## Appendix 2

### Chemical similarity between substrates associated with GO terms

#### Mapping the Gene Ontology to ChEBI

Cross-ontology relations between GO and the ChEBI ontology [39] were retrieved through the QuickGO API [40]. After the preprocessing described in Section Dataset creation pipeline, the GO terms are mapped to their corresponding ChEBI terms, if any are available.

#### Calculating the chemical similarity of two GO terms

In order to calculate a measure of the chemical similarity between two substrate transport GO terms, the GO annotations are first filtered for those that are annotated with ChEBI identifiers representing primary substrate molecules. Molecular fingerprints are derived from the SMILES-representations [41] of the ChEBI terms, using RDKit [42]. Four different methods are used: Morgan [43], Atompairs [44], Topological torsion [45], and MACCS [46]. The chemical similarity between two substrates is estimated as the Tanimoto coefficient [47] of their fingerprints.

The chemical similarity between two substrate-transport GO terms is then defined as the Tanimoto coefficient between the respective transported substrates. If more than one molecule is associated with a GO term, the average, maximal and minimal pairwise Tanimoto coefficient is calculated.

#### Results on yeast transporter dataset

We explored if ChEBI terms associated with GO terms can be used to filter out non-substrate related transporter function GO terms, such as those that describe transport mechanisms. Of these 288 GO terms, 221 were annotated with ChEBI terms, and 149 of which had SMILES representations available. Only 15 GO terms had both SMILES representations of their primary substrates, and were annotated with 20 or more proteins from yeast. These 15 GO terms annotate 173 of 332 transmembrane transport proteins in *S. cerevisiae*, meaning that only using GO terms that are annotated with ChEBI terms would lower the number of proteins and substrate classes in our dataset considerably.

## Appendix 3

### Transporter dataset pipeline parameters

#### Manually curated proteins

Proteins that have not been manually curated (i.e. those available in TrEMBL but not in SwissProt) can be removed from the dataset.

#### Sequence evidence

Proteins with experimental sequence evidence at transcript level but not at protein level can be added to the dataset. Predicted sequences are removed during pre-processing, and are not available for the pipeline.

#### GO evidence

GO annotations can be filtered for their evidence codes, e.g. to remove electronically inferred information that has not been manually reviewed. Annotations with the NOT prefix, which denotes that a protein is explicitly *not* annotated with a particular GO term, are already removed during pre-processing.

#### Organisms

Proteins can be filtered for a subset of taxonomy identifiers, e.g. 9606 for human.

#### External proteins

Proteins that do not occur in the provided organisms can be added to the dataset, e.g. to simulate experiments where proteins from one organism are expressed in another one.

#### Gene names available

The 671,750 proteins without any annotated gene names can be removed from the dataset, for example if the data needs to be linked to genetics- or transcriptomics datasets.

#### GO subset

A sub-tree of GO can be selected by providing a new root node. Further filtering can be applied to the GO subset, for example for the aspect (i.e. *molecular_function*, *cellular_component*, *biological_process*) and for a subset of edge annotations (relationships) between GO terms (e.g. *is_a*). All GO terms that are not descendants of the new root node, according to the filtered subset of edge annotations, are then removed from the graph. Only proteins annotated with this subset of GO terms remain in the dataset.

#### ChEBI quality

ChEBI terms can optionally be filtered by their star rating system, e.g. for those that have been manually verified.

#### Cellular component

Optionally, a subset of cellular component annotations can be created, which then get filtered for those with the *located_in* or *is_active_in* qualifiers, and only *is_a* relations between GO terms are kept. Proteins are also annotated with the ancestor GO terms of their cellular components. The subset of transport proteins that are located in a particular set of compartments, such as *plasma membrane*, *mitochondrion* or *eisosome*, can then be selected for further analysis.

## Appendix 4

### Steps in the algorithm pipeline

**Input**.

- Transport protein dataset and corresponding GO annotation data created with the transporter dataset creation pipeline described in Section Dataset creation pipeline
- Ontology graph for the annotation data in OBO format, created by the preprocessing pipeline (see Section Data retrieval and preprocessing)

**Parameters**.

- **n** GO terms annotated with fewer than n proteins in the dataset are removed. (Default: 20)
- **m** GO terms annotated with fewer than m unique proteins are removed, i.e. proteins that are only annotated with one of the GO terms in a binary classification task, not both. (Default: 15)
- **p** The top pth percentile of GO terms according to the distribution of annotated samples is removed (see Section Greedy algorithm for clustering of GO terms). (Default: 0)
- **c** The minimum allowed coverage of the resulting set of GO terms, i.e. the percentage of proteins in the original protein dataset that are annotated with a GO term from the optimized subset. (Default: 0.9)
- **a** Whether to prefer abstract or specific GO terms. Higher specificity is defined as increased distance to root node. (Default: specific)
- ***ϵ*** Reduces the influence of rounding errors and local minima on the algorithm, by allowing the optimization to consider GO terms that are within *ϵ* of the best score during the iteration. Scores are in the interval [0, 1], therefore values of *ϵ* < = 0.05 are recommended. (Default: 0.0)

**Steps**.

1. Create a starting set of GO terms to optimize from the provided proteins and their annotations.
2. Filter the GO terms in the set, as well as the corresponding ontology graph, according to parameters *n*, *m* and *p*.
3. Train and evaluate pairwise binary ML models for GO terms, as described in Section Evaluation of pairwise machine learning models
4. For each GO term calculate:
  (a) Coverage loss: The percentage of protein that would be annotated if the GO term was removed from the current set.
  (b) Score delta: The average ML evaluation score across all pairwise models, if the GO term was removed from the current set.
5. Removal candidates: Select all GO terms that can be removed without the coverage falling below *c*.
6. Sort removal candidates by score delta, select those with the highest improvement in average F1 score when removed, while also considering those that are within *ϵ* of the maximum score.
7. End condition: If the list of removal candidates is empty then return the optimized list of GO terms.
8. If the list of removal candidates contains more than one GO term:
  1. Select either the most specific or most abstract term(s), depending on parameter *a*.
  2. If the list of removal candidates still contains more than one GO term, draw one of them using deterministic random sampling.
9. Remove the selected GO term from the set, go back to step 4 and repeat the process with the smaller subset.

## Supporting information

S1 Fig Overlap percentage heatmap for yeast dataset at 98% coverage. Heatmap showing the fraction of yeast dataset proteins annotated with GO term 1 that are also annotated with GO term 2, for all pairs of GO terms in the optimized subset. Removal of any term would cause the protein coverage to fall below the specified 98%.
(TIF)

S2 Fig Median F1 scores for meta-organism dataset when not removing abstract terms. Median F1 scores between pairs of GO terms for the meta-organism dataset, when not removing the top 5th percentile of GO terms according to sample count.
(TIF)

S3 Fig Subset sizes for meta-organism dataset when not removing abstract terms. Final subset sizes for the meta-organism dataset after applying the pipeline, when not removing the top 5th percentile of GO terms according to sample count.
(TIF)

S4 Fig GO terms that were excluded in meta-organism dataset due to low sample count, when not removing abstract terms. Number of GO terms with no available F1 scores for the meta-organism dataset, when not removing the top 5th percentile of GO terms according to sample count.
(TIF)

S5 Fig GO terms excluded in yeast dataset due to low sample count, at different thresholds. Number of GO term pairs in the yeast dataset without evaluation scores available, at different protein coverage thresholds and for four different values of *m*. ML models are only available for pairs that are distinct enough, meaning that each term has at least *m* proteins available for training that are not also annotated with the respective other term.
(TIF)

S6 Fig Median F1 scores for yeast dataset, at different thresholds for coverage and sample count. Median F1 scores between pairs of GO terms in the yeast dataset, at different protein coverage thresholds, using four different evaluation matrices that were created with different values of *m*, i.e. the threshold for how few unique samples are allowed per class during training.
(TIF)

S7 Fig Optimized subset sizes for yeast dataset, at different thresholds for coverage and sample count. Subset sizes found by the redundancy reduction pipeline for GO term subsets in the yeast dataset, at different values of *m*. With lower coverage, fewer terms are necessary for reaching the threshold. At higher values of *m*, there are more pairs in the dataset with no ML model available, and these terms are removed first by the pipeline.
(TIF)

S8 Fig Overlap percentage heatmap for the meta-organism dataset, after removing the most abstract GO terms. Heatmap showing of the fraction of proteins annotated with GO term 1 that is also annotated with GO term 2, for all pairs of GO terms in the optimized subset for the meta-organism dataset, when removing the top 5th percentile of GO terms. Here, the coverage threshold was reduced to 67, and *m* was set to 20.
(TIF)

S9 Fig Overlap percentage heatmap for the meta-organism dataset, when using the *ϵ* parameter at 99% coverage. Heatmap showing the fraction of proteins annotated with GO term 1 that is also annotated with GO term 2, for all pairs of GO terms in the optimized subset. This subset was generated by preferring abstract GO terms and setting *ϵ* to 0.005. Removal of any term would cause the protein coverage to fall below the specified 99%.
(TIF)

S10 Fig Overlap percentage heatmap for the meta-organisn dataset, when not removing the most abstract GO terms. Heatmap showing the fraction of proteins annotated with GO term 1 that are also annotated with GO term 2, for all pairs of GO terms in the optimized subset for the meta-organism dataset, but without removing the top 5th percentile of GO terms.
(TIF)

S11 Fig Flowchart of the entire pipeline. First, the raw data is filtered and cleaned, and converted to a binary data format for faster reading (see Section Data retrieval and preprocessing). Next, the general protein dataset is converted to a specific transporter dataset according to specified parameters (see Section Dataset creation pipeline). Then, the protein feature generation algorithms described in our previous study [22] are applied to the data, and pairwise ML models are trained and evaluated (see Section Evaluation of pairwise machine learning models). Finally, the iterative optimization algorithm described in Section Greedy algorithm for clustering of GO terms and in Appendix 4 is applied to the dataset, and an optimized set of functional annotations is returned.
(PNG)

S1 Table Meta dataset protein counts. Number of unique proteins in the transmembrane transporter dataset, after filtering for criteria related to data quality.
(PDF)

S2 Table Meta dataset GO term counts. Number of unique GO terms in the transmembrane transporter dataset, after filtering for criteria related to data quality.
(PDF)

S3 Table Yeast dataset pairwise evaluation scores. F1 scores for different machine learning models that were trained on pairs of GO terms and their associated proteins, optionally with 70% sequence clustering, for the yeast dataset. The goal was to remove the worst-performing GO terms with the clustering pipeline described in Section Greedy algorithm for clustering of GO terms. ANOVA and PCA refer to two different types methods that were used to reduce the number of feature dimensions (see Section scoresEvaluation of pairwise machine learning models).
(PDF)
