# Supplementary figures and images for: Identifying optimal substrate classes of membrane transporters
Source: PLoS One. 2024 Dec 19;19(12):e0315330. doi: 10.1371/journal.pone.0315330 (PMC11658592; doi:10.1371/journal.pone.0315330)

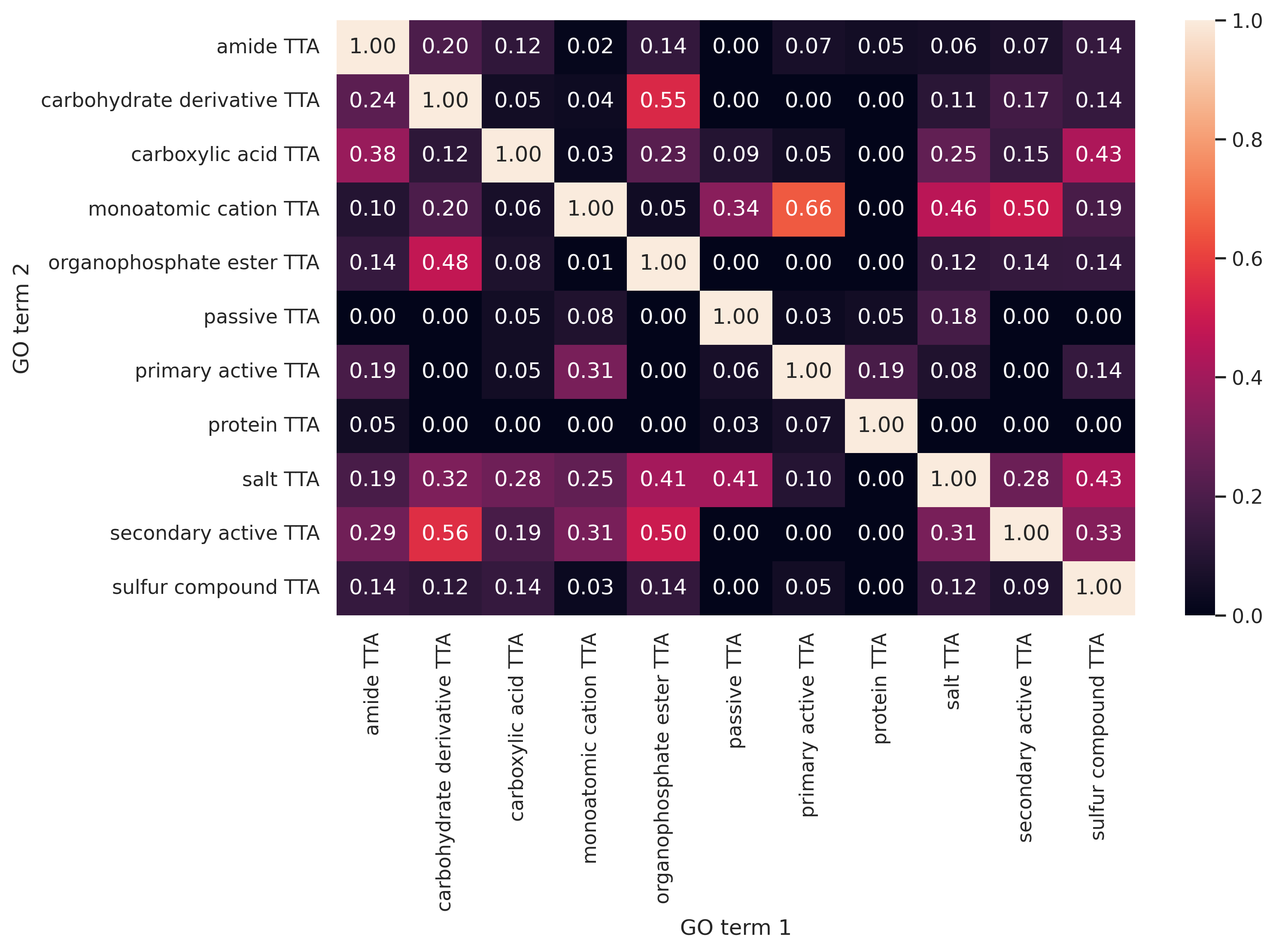

Supplement: S1 Fig — Heatmap showing the fraction of yeast dataset proteins annotated with GO term 1 that are also annotated with GO term 2, for all pairs of GO terms in the optimized subset. Removal of any term would cause the protein coverage to fall below the specified 98%. (TIF) [file pone.0315330.s001.tif]

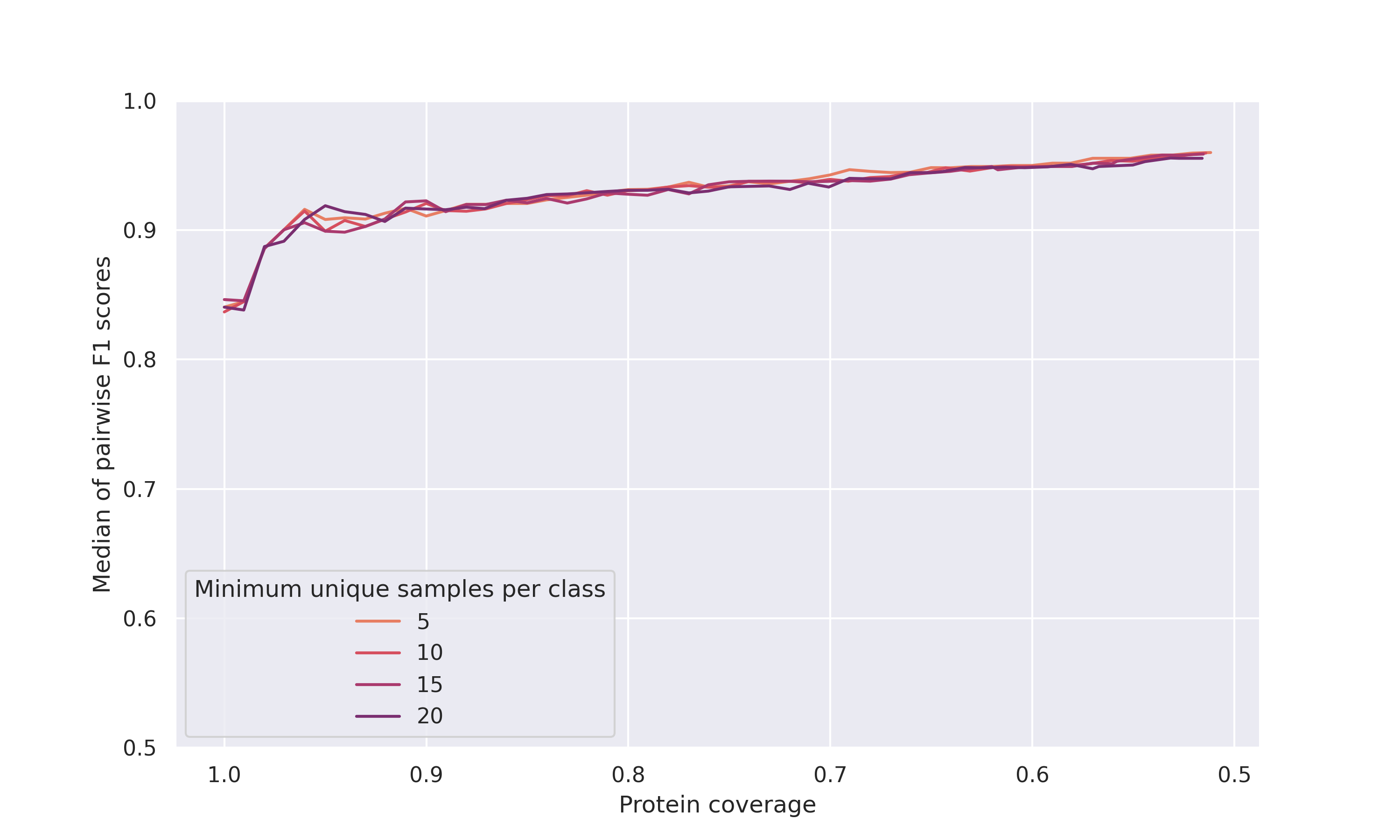

Supplement: S2 Fig — Median F1 scores between pairs of GO terms for the meta-organism dataset, when not removing the top 5th percentile of GO terms according to sample count. (TIF) [file pone.0315330.s002.tif]

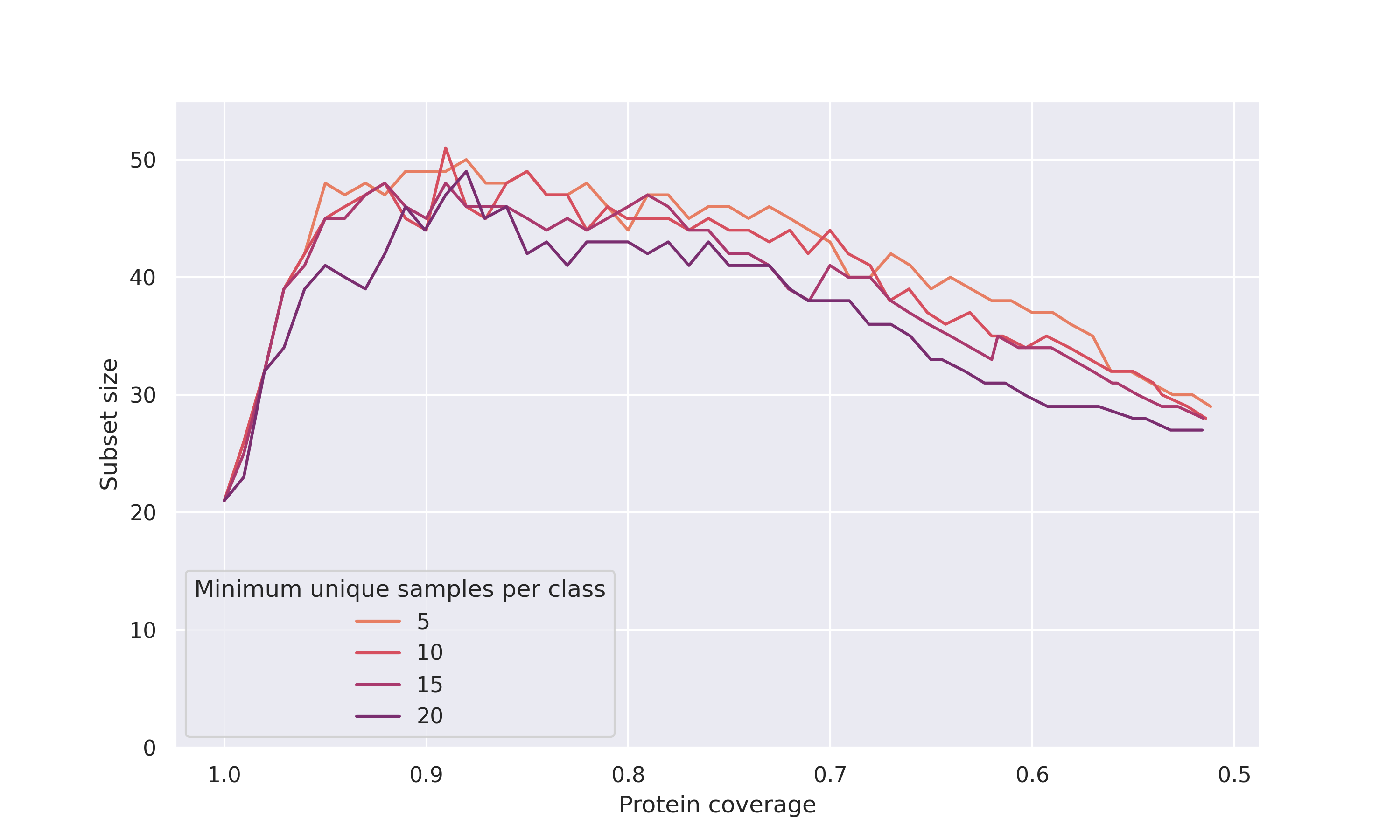

Supplement: S3 Fig — Final subset sizes for the meta-organism dataset after applying the pipeline, when not removing the top 5th percentile of GO terms according to sample count. (TIF) [file pone.0315330.s003.tif]

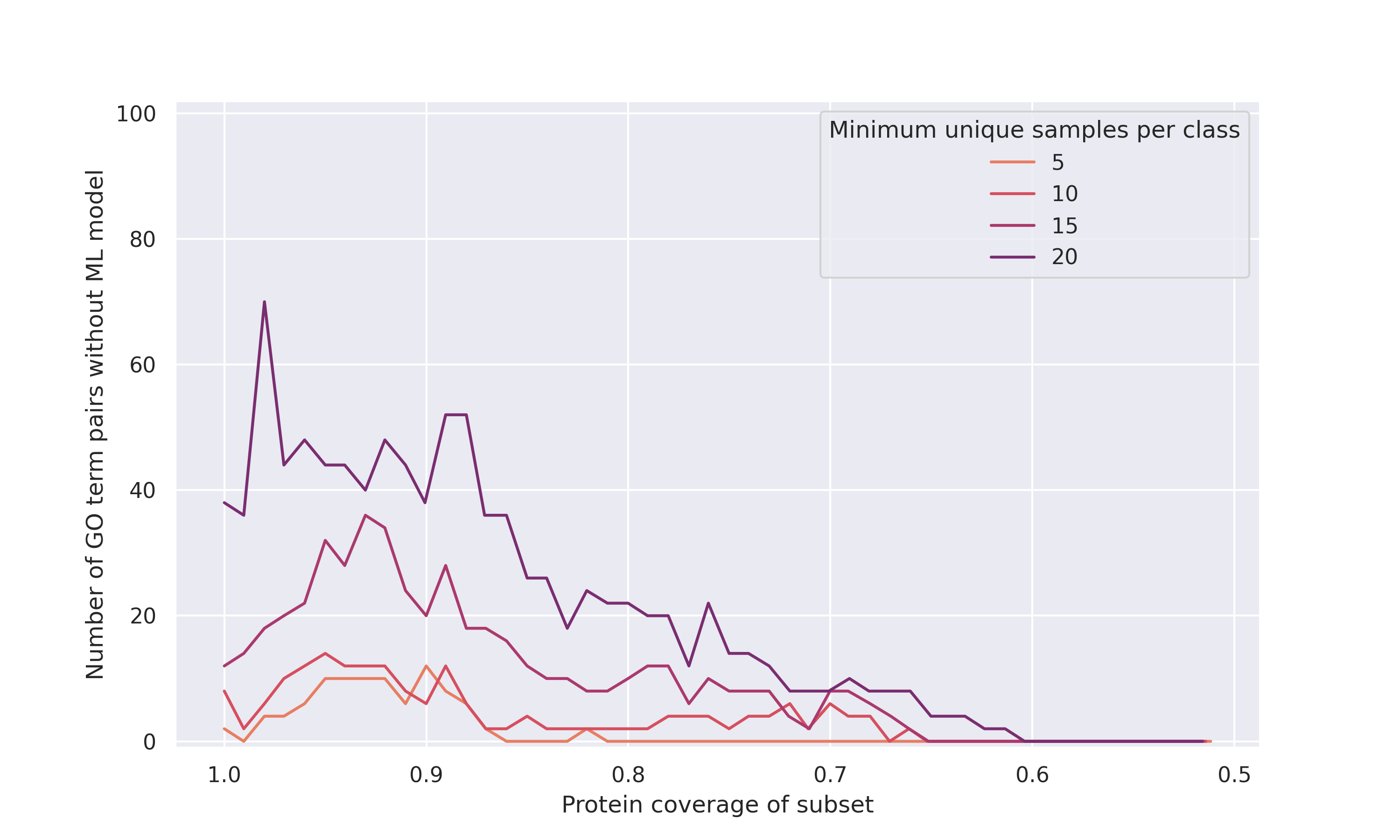

Supplement: S4 Fig — Number of GO terms with no available F1 scores for the meta-organism dataset, when not removing the top 5th percentile of GO terms according to sample count. (TIF) [file pone.0315330.s004.tif]

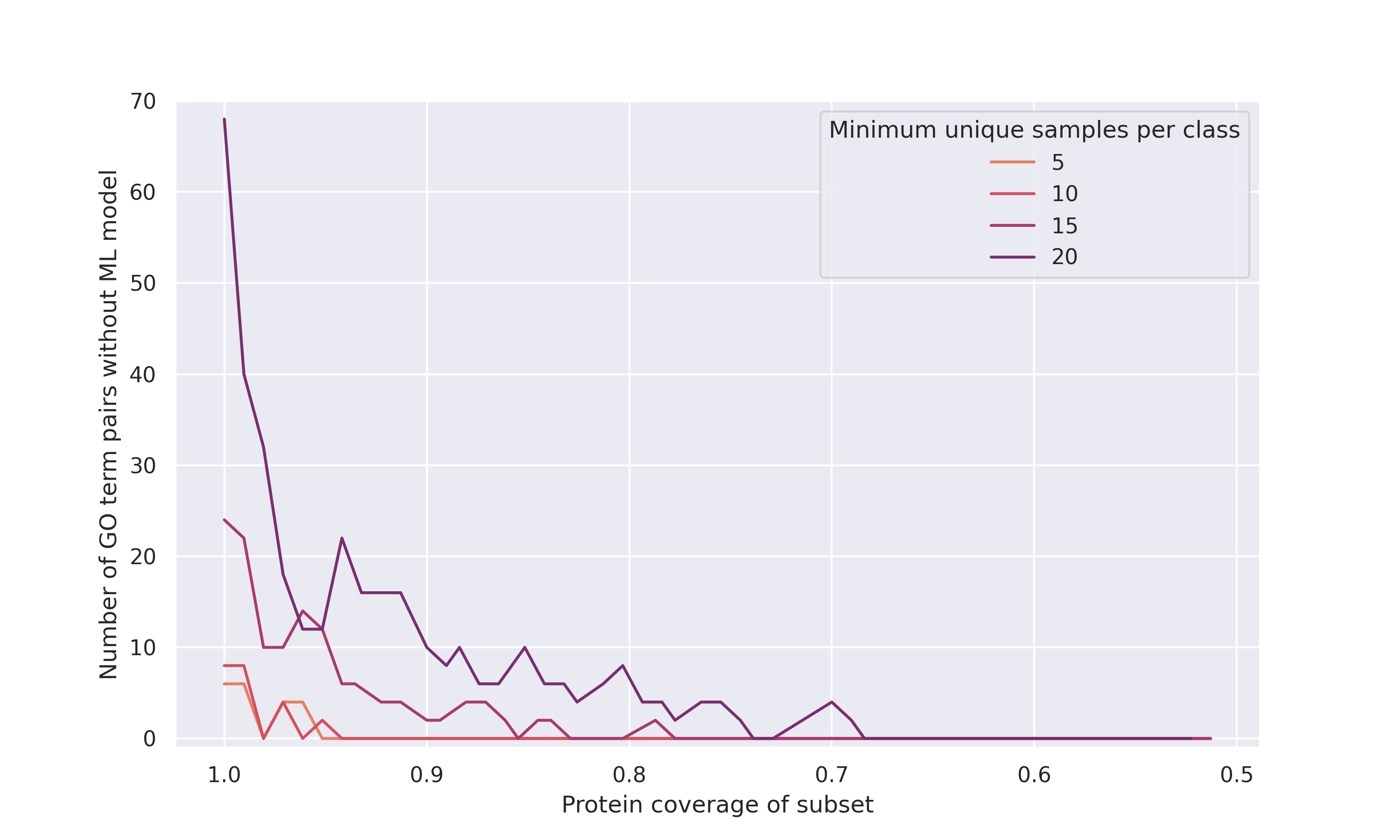

Supplement: S5 Fig — Number of GO term pairs in the yeast dataset without evaluation scores available, at different protein coverage thresholds and for four different values of m. ML models are only available for pairs that are distinct enough, meaning that each term has at least m proteins available for training that are not also annotated with the respective other term. (TIF) [file pone.0315330.s005.tif]

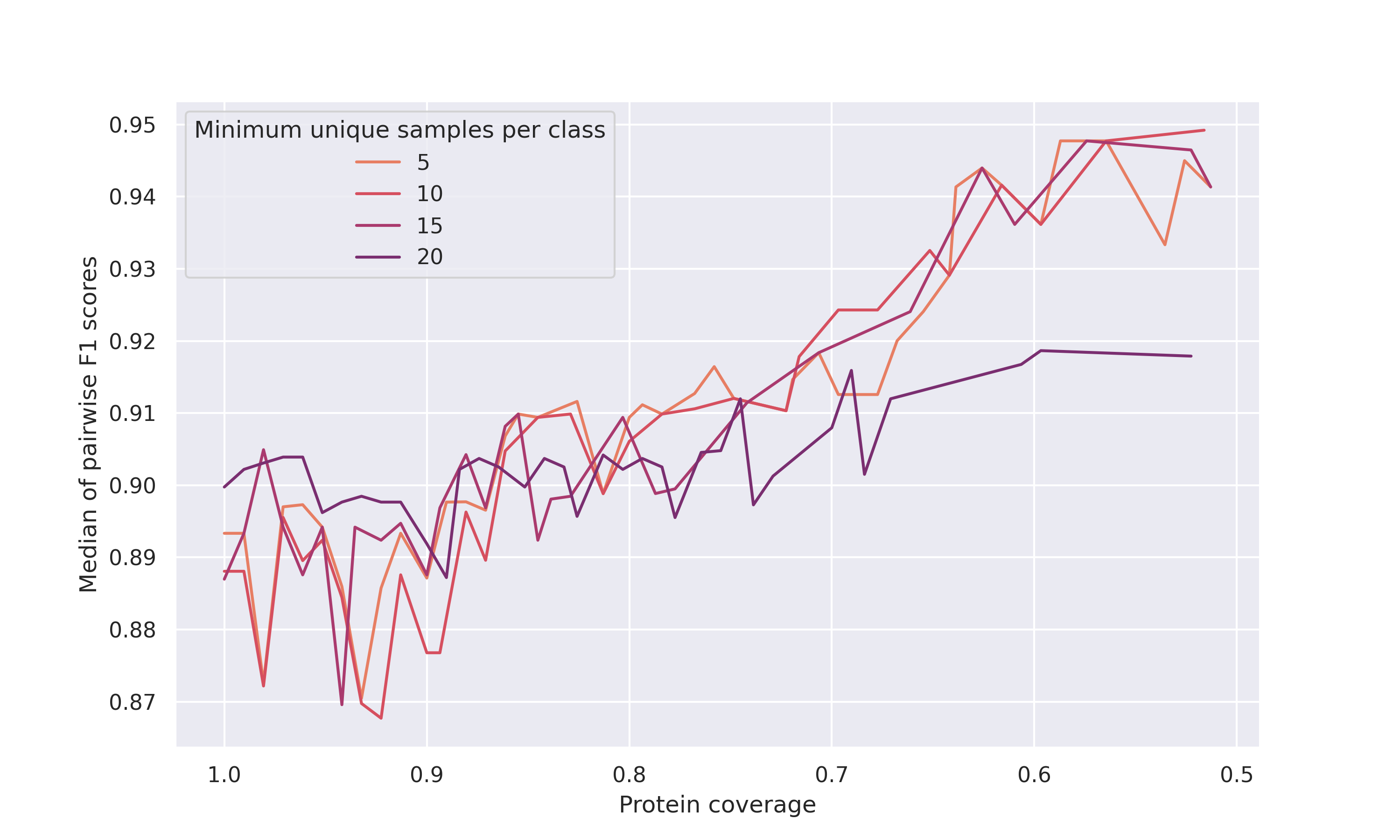

Supplement: S6 Fig — Median F1 scores between pairs of GO terms in the yeast dataset, at different protein coverage thresholds, using four different evaluation matrices that were created with different values of m, i.e. the threshold for how few unique samples are allowed per class during training. (TIF) [file pone.0315330.s006.tif]

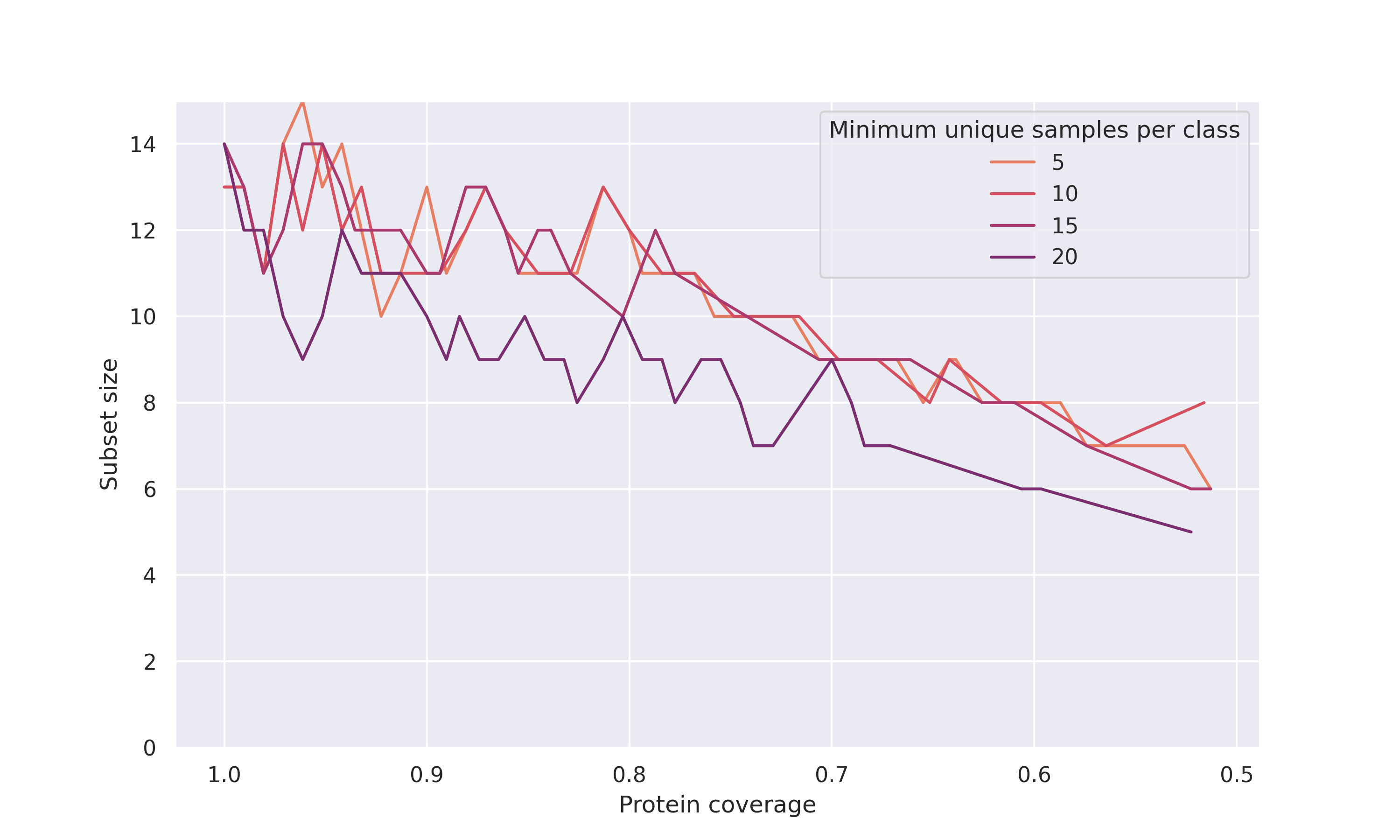

Supplement: S7 Fig — Subset sizes found by the redundancy reduction pipeline for GO term subsets in the yeast dataset, at different values of m. With lower coverage, fewer terms are necessary for reaching the threshold. At higher values of m, there are more pairs in the dataset with no ML model available, and these terms are removed first by the pipeline. (TIF) [file pone.0315330.s007.tif]

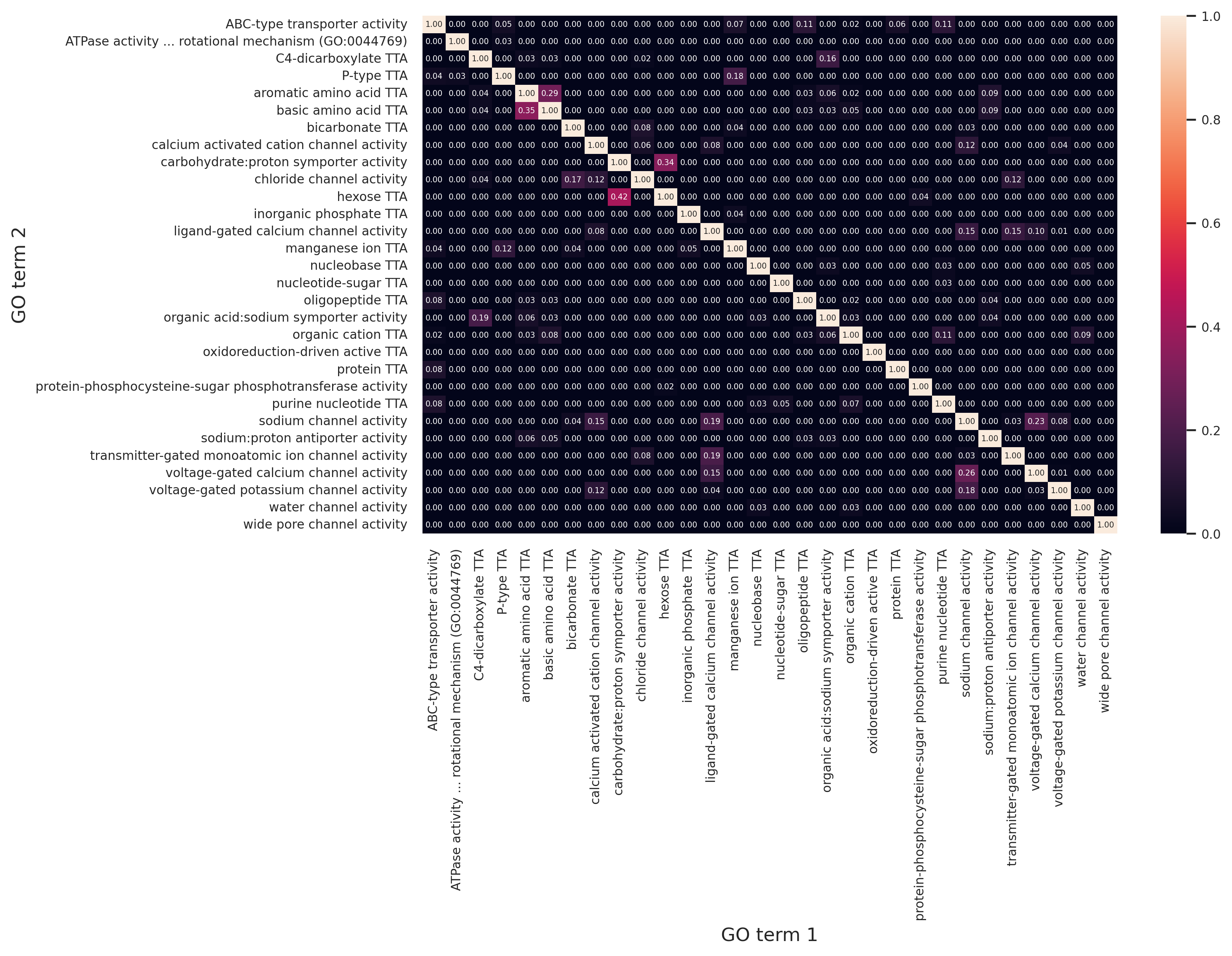

Supplement: S8 Fig — Heatmap showing of the fraction of proteins annotated with GO term 1 that is also annotated with GO term 2, for all pairs of GO terms in the optimized subset for the meta-organism dataset, when removing the top 5th percentile of GO terms. Here, the coverage threshold was reduced to 67, and m was set to 20. (TIF) [file pone.0315330.s008.tif]

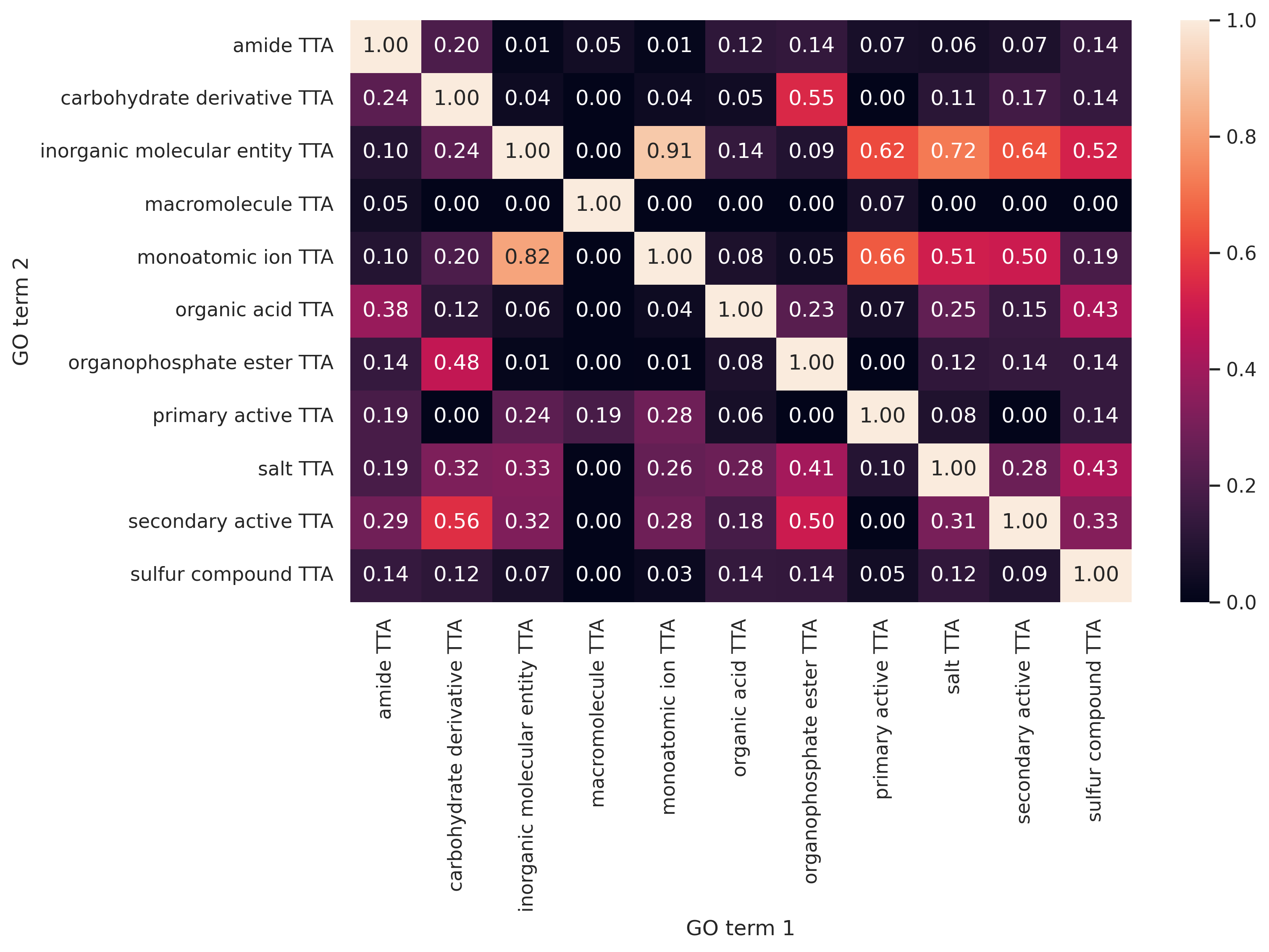

Supplement: S9 Fig — Heatmap showing the fraction of proteins annotated with GO term 1 that is also annotated with GO term 2, for all pairs of GO terms in the optimized subset. This subset was generated by preferring abstract GO terms and setting ϵ to 0.005. Removal of any term would cause the protein coverage to fall below the specified 99%. (TIF) [file pone.0315330.s009.tif]

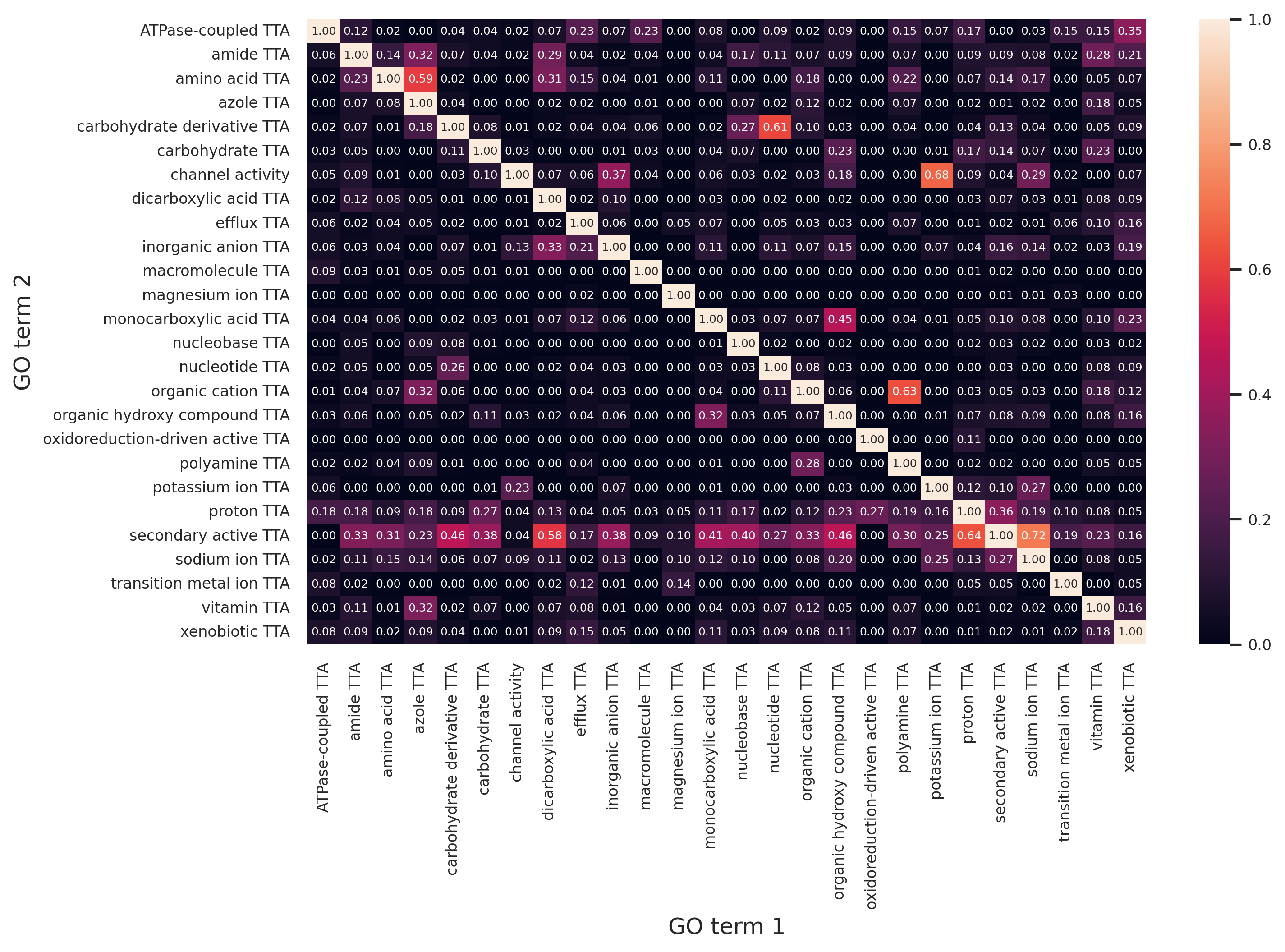

Supplement: S10 Fig — Heatmap showing the fraction of proteins annotated with GO term 1 that are also annotated with GO term 2, for all pairs of GO terms in the optimized subset for the meta-organism dataset, but without removing the top 5th percentile of GO terms. (TIF) [file pone.0315330.s010.tif]

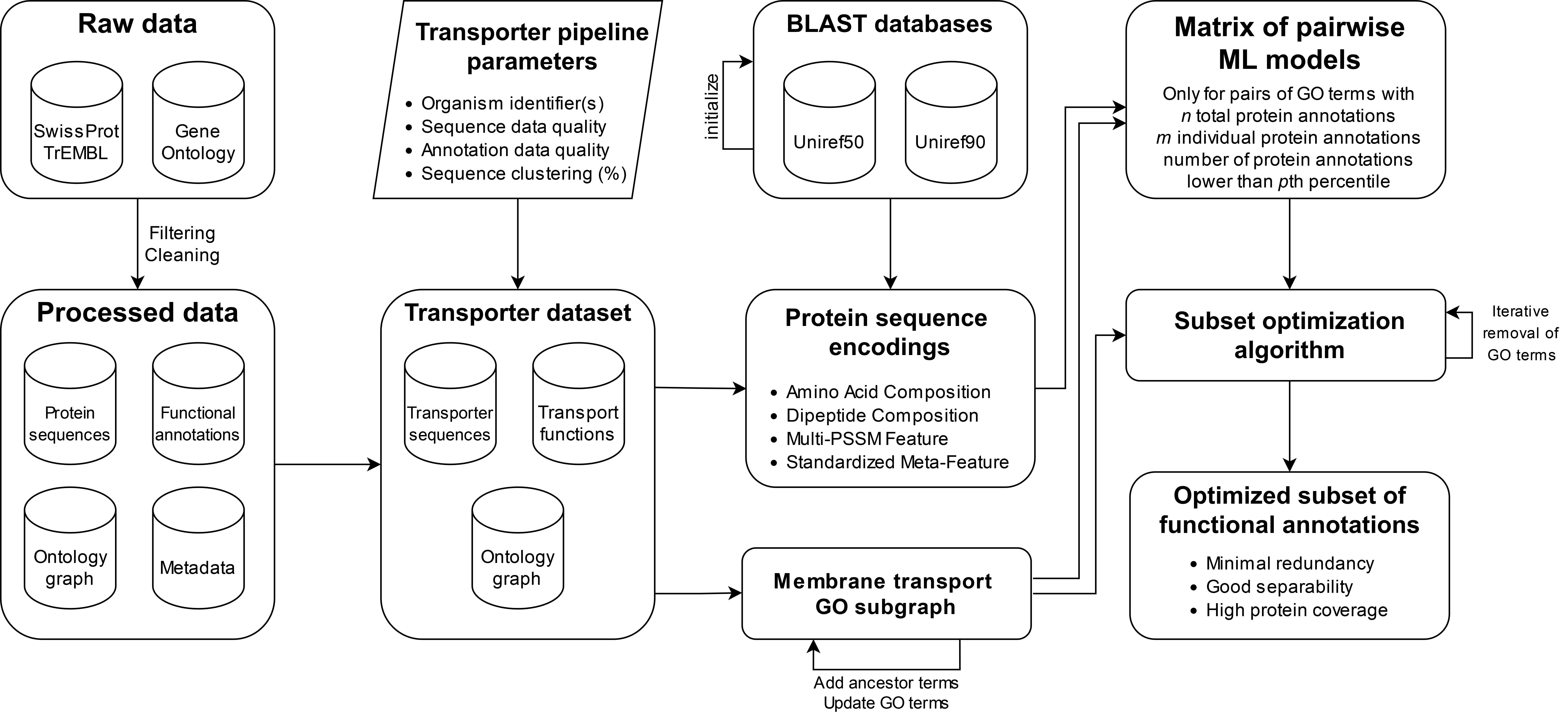

Supplement: S11 Fig — First, the raw data is filtered and cleaned, and converted to a binary data format for faster reading (see Section Data retrieval and preprocessing). Next, the general protein dataset is converted to a specific transporter dataset according to specified parameters (see Section Dataset creation pipeline). Then, the protein feature generation algorithms described in our previous study [22] are applied to the data, and pairwise ML models are trained and evaluated (see Section Evaluation of pairwise machine learning models). Finally, the iterative optimization algorithm described in Section Greedy algorithm for clustering of GO terms and in Appendix 4 is applied to the dataset, and an optimized set of functional annotations is returned. (PNG) [file pone.0315330.s011.png]
