# Supplementary material for: Identifying optimal substrate classes of membrane transporters
Source: PLoS One. 2024 Dec 19;19(12):e0315330. doi: 10.1371/journal.pone.0315330 (PMC11658592; doi:10.1371/journal.pone.0315330)
Supplement: S1 Table — Number of unique proteins in the transmembrane transporter dataset, after filtering for criteria related to data quality. (PDF) [file pone.0315330.s012.pdf]

Swissprot reviewed	Known gene	GO evidence	Protein evidence	Cluster thresh.[%]	Four organisms	Proteins Uniprot
True	True	experiment	protein level	50	1313	1331
				70	1602	1852
				90	1779	2678
				100	1892	3919
				None	1915	4022
			transcript level	50	270	449
				70	384	808
				90	487	1385
				100	521	2092
				None	532	2147
		computational	protein level	50	1185	1649
				70	1492	2391
				90	1664	3415
				100	1793	4824
				None	1821	4990
			transcript level	50	280	585
				70	426	1087
				90	554	1880
				100	592	2868
				None	607	2989
False	True	experiment	protein level	50	24	392
				70	26	573
				90	31	841
				100	34	1279
				None	34	1374
			transcript level	50	1	430
				70	2	813
				90	3	1424
				100	6	2344
				None	7	2538
		computational	protein level	50	188	905
				70	262	1426
				90	456	2211
				100	1080	4373
				None	1294	5016
			transcript level	50	44	3263
				70	65	6228
				90	119	11625
				100	538	24217
				None	789	27705
	False	experiment	transcript level	50	1	18
				70	1	37
				90	1	81
				100	2	116
				None	3	117
		computational	protein level	50	1	19
				70	1	30
				90	5	58
				100	9	181
				None	9	185
			transcript level	50	61	3820
				70	77	7133
				90	145	12032
				100	648	19559
				None	751	21208

Table S1
