# Supplementary material for: Identifying optimal substrate classes of membrane transporters
Source: PLoS One. 2024 Dec 19;19(12):e0315330. doi: 10.1371/journal.pone.0315330 (PMC11658592; doi:10.1371/journal.pone.0315330)
Supplement: S2 Table — Number of unique GO terms in the transmembrane transporter dataset, after filtering for criteria related to data quality. (PDF) [file pone.0315330.s013.pdf]

| | | | | | Transporter GO terms | |
|---|---|---|---|---|---|---|
| | | | | | Four organisms | Uniprot |
| Swissprot reviewed | Known gene | GO evidence | Protein evidence | Cluster thresh.[%] | | |
| True | True | experiment | protein level | 50 | 650 | 581 |
| | | | | 70 | 679 | 639 |
| | | | | 90 | 693 | 711 |
| | | | | 100 | 702 | 746 |
| | | | | None | 705 | 747 |
| | | | transcript level | 50 | 268 | 349 |
| | | | | 70 | 300 | 420 |
| | | | | 90 | 314 | 480 |
| | | | | 100 | 319 | 540 |
| | | | | None | 322 | 542 |
| | | computational | protein level | 50 | 449 | 390 |
| | | | | 70 | 492 | 462 |
| | | | | 90 | 499 | 546 |
| | | | | 100 | 509 | 616 |
| | | | | None | 509 | 617 |
| | | | transcript level | 50 | 166 | 232 |
| | | | | 70 | 189 | 282 |
| | | | | 90 | 197 | 337 |
| | | | | 100 | 203 | 440 |
| | | | | None | 205 | 443 |
| False | True | experiment | protein level | 50 | 62 | 299 |
| | | | | 70 | 64 | 320 |
| | | | | 90 | 67 | 347 |
| | | | | 100 | 70 | 388 |
| | | | | None | 70 | 389 |
| | | | transcript level | 50 | 17 | 261 |
| | | | | 70 | 19 | 336 |
| | | | | 90 | 22 | 372 |
| | | | | 100 | 30 | 397 |
| | | | | None | 43 | 400 |
| | | computational | protein level | 50 | 157 | 261 |
| | | | | 70 | 176 | 299 |
| | | | | 90 | 188 | 360 |
| | | | | 100 | 207 | 441 |
| | | | | None | 213 | 447 |
| | | | transcript level | 50 | 98 | 254 |
| | | | | 70 | 101 | 311 |
| | | | | 90 | 112 | 373 |
| | | | | 100 | 147 | 502 |
| | | | | None | 195 | 520 |
| | False | experiment | transcript level | 50 | 8 | 56 |
| | | | | 70 | 8 | 90 |
| | | | | 90 | 8 | 131 |
| | | | | 100 | 8 | 153 |
| | | | | None | 23 | 153 |
| | | computational | protein level | 50 | 5 | 32 |
| | | | | 70 | 5 | 41 |
| | | | | 90 | 28 | 64 |
| | | | | 100 | 28 | 80 |
| | | | | None | 28 | 80 |
| | | | transcript level | 50 | 118 | 215 |
| | | | | 70 | 119 | 247 |
| | | | | 90 | 123 | 277 |
| | | | | 100 | 186 | 302 |
| | | | | None | 196 | 306 |

Table S2
