# Supplementary material for: Identifying optimal substrate classes of membrane transporters
Source: PLoS One. 2024 Dec 19;19(12):e0315330. doi: 10.1371/journal.pone.0315330 (PMC11658592; doi:10.1371/journal.pone.0315330)
Supplement: S3 Table — F1 scores for different machine learning models that were trained on pairs of GO terms and their associated proteins, optionally with 70% sequence clustering, for the yeast dataset. The goal was to remove the worst-performing GO terms with the clustering pipeline described in Section Greedy algorithm for clustering of GO terms. ANOVA and PCA refer to two different types methods that were used to reduce the number of feature dimensions (see Section scoresEvaluation of pairwise machine learning models). (PDF) [file pone.0315330.s014.pdf]

Clustering	Dataset	Method	min	mean	median	max	sdev
None	train	None	0.11	0.86	0.90	0.99	0.14
		ANOVA	0.41	0.88	0.91	1.00	0.11
		PCA	0.37	0.87	0.90	1.00	0.10
	test	None	0.15	0.87	0.91	0.99	0.13
		ANOVA	0.37	0.88	0.90	1.00	0.11
		PCA	0.23	0.87	0.90	1.00	0.12
70%	train	None	0.12	0.83	0.87	0.99	0.14
		ANOVA	0.45	0.85	0.88	0.99	0.11
		PCA	0.38	0.84	0.87	0.97	0.11
	test	None	0.13	0.84	0.88	0.98	0.14
		ANOVA	0.42	0.84	0.87	0.98	0.11
		PCA	0.26	0.84	0.87	0.98	0.11

Table S3
